# Piezo1 regulates remodeling of skin-derived extracellular matrix by embedded umbilical cord mesenchymal stem cells in a stiffness-dependent fashion

**DOI:** 10.1016/j.mtbio.2026.102883

**Published:** 2026-01-31

**Authors:** Fenghua Zhao, Xue Zhang, Theo Borghuis, Linda A. Brouwer, Janette K. Burgess, Prashant K. Sharma, Martin C. Harmsen

**Affiliations:** a University of Groningen, University Medical Centre Groningen, Department of Biomaterials and Biomedical Technology-FB40, A. Deusinglaan 1, Groningen, 9713 AV, the Netherlands; b University of Groningen, University Medical Centre Groningen, Department of Pathology and Medical Biology, Hanzeplein 1 (EA11), Groningen, 9713 GZ, the Netherlands; c University of Groningen, University Medical Centre Groningen, Groningen Research Institute for Asthma and COPD (GRIAC), Hanzeplein 1 (EA11), Groningen, 9713 AV, the Netherlands

**Keywords:** Mechanotransduction, Piezo1, UC-MSCs, ECM remodeling, Stiffness, Fibrotic environments

## Abstract

Cells continuously sense and adapt to the mechanical properties of their surrounding extracellular matrix (ECM), yet how human umbilical cord–derived mesenchymal stromal cells (UC-MSCs) mechanotransduce stiffness cues in 3D ECM remains incompletely understood. This knowledge gap limits the rational design of MSC-based regenerative therapies and mechanically instructive biomaterials. Here, using ruthenium-catalyzed photocrosslinked skin-derived ECM hydrogels spanning a physiological to fibrotic stiffness range, we demonstrate that UC-MSCs exhibit distinct, stiffness-dependent remodeling strategies. Soft matrices (1.2 kPa) induced cell-mediated hydrogel contraction, medium stiffness (3.4 kPa, comparable to native skin) supported elongated cell morphology with minimal remodeling, whereas stiff matrices (17.7 kPa) kept seeded UC-MSCs rounded and induced pericellular void formation consistent with localized ECM remodeling. By decoupling geometric contraction from intrinsic ECM turnover using volume-normalized mechanical analyses, we identify the Piezo1 as a key regulator of stiffness-dependent adaptation. Piezo1 expression increased with stiffness, and its inhibition attenuated contraction in soft matrices and prevented stiffness reduction in stiff matrices, indicating that Piezo1 enables MSCs to mechanically adapt across 3D microenvironments. Analysis of matrix metalloproteinase expression revealed stiffness-dependent regulation of MMP2 and MMP14; however, their expression was only marginally affected by Piezo1 inhibition, suggesting that Piezo1 influences ECM remodeling through mechanisms beyond direct regulation of MMP expression. Together, these findings establish a mechanistic framework in which UC-MSCs adapt to 3D ECM stiffness through Piezo1-dependent mechanosensing. This work provides conceptual and practical guidance for the design of mechanically programmable biomaterials, the optimization of MSC-based regenerative strategies, and therapeutic approaches aimed at modulating pathological tissue mechanics such as fibrosis.

## Highlights

• Stiffness-tunable ECM hydrogels mimic stiffnesses of healthy skin and scar.
• Embedded UC-MSCs remodel ECM hydrogels in a stiffness-dependent manner.
• Piezo1 expression in UC-MSCs increases with ECM stiffness.
• Piezo1 inhibition disrupts stiffness-specific contraction and ECM degradation.
• This study provides novel insights into UC-MSC responses to fibrotic environments, informing regenerative strategies.

## Graphical abstract

**Figure:**
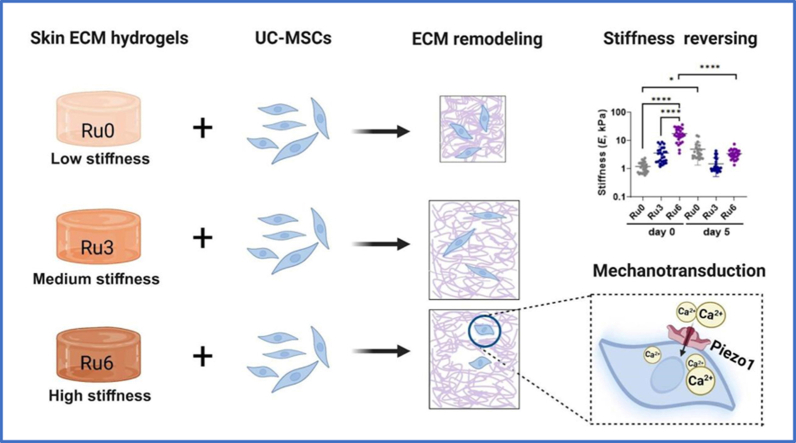

## 1 Introduction

The extracellular matrix (ECM) provides mechanical and biochemical cues that profoundly influence cell fate and function [1,2]. The mechanical properties of ECM and its influence on adhered cells, is (mechano)sensed and relayed downstream via mechanotransduction signaling pathways. These processes may cause phenotypic changes which, among others, result in remodeling of the ECM by the embedded cells. Remodeling is the net result of degradation of existing ECM and deposition of new ECM, while cells may also physically recombine existing fiber structures. Thus, the relationship between ECM and cells is reciprocal and dynamic. Yet, the molecular and physical understanding of this reciprocity is in its infancy.

Mesenchymal cells, such as fibroblasts and mesenchymal stromal cells (MSCs), are professional ECM remodelers. Our previous studies demonstrated that matrix stiffness regulates the expression of key ECM remodeling enzymes, such as matrix metalloproteinases (MMPs), in adipose tissue-derived MSCs and fibroblasts. Their degrading of ECM is part of the process to establish an optimal i.e. homeostatic mechanical microenvironment [[3], [4], [5]]. In a clinical and pre-clinical setting, human MSCs, such as umbilical cord-derived MSCs (UC-MSCs), were shown to facilitate injury repair and reduce, if not reverse, fibrosis. Much of this therapeutic effect is governed by their secretory profile and ECM remodeling activity [6].

Considering the ECM is a mechanical support framework, it stands to reason that virtually all cellular contacts with surrounding ECM participate in mechanosensing and subsequent mechanotransduction. While ECM composition is organ and tissue-dependent and cellular adhesions such as integrin-dependent focal adhesions are cell-dependent, mechanical regulation is ECM and cell-dependent too [7]. One of the best-studied mechanoresponsive pathways is Hippo with its main downstream mediators YAP/TAZ (Yes-associated protein (YAP)/transcriptional coactivator with PDZ-binding motif (TAZ)) [[8], [9], [10]]. In general, a high stiffness causes nuclear translocation of YAP/TAZ and activation of mechanoresponsive genes. Besides this pathway, ion channels physically respond to mechanical stimulation by altering ion fluxes. Although best-studied in neurons, the calcium channel Piezo1 is a mechanosensor that is activated by mechanical forces such as stretch, shear stress, and ECM stiffness [[11], [12], [13], [14]]. In fact, Piezo1 appears to be augmenting ECM-based diseases such as fibrosis where excessive ECM deposition increases matrix stiffness [15]. In experimental unilateral ureteral obstruction (UUO)-induced renal fibrosis, the increased ECM deposition and stiffness activate Piezo1 in renal mesangial cells. This is also observed in clinical kidney fibrosis where Piezo1 is activated in proximal tubular cells and collecting duct epithelial cells [16]. Additionally, in vitro stimulation of Piezo1 using Yoda1, a small-molecule agonist of Piezo1, induced calcium influx and initiated profibrotic responses in human proximal tubular cells [17]. In fibrotic human and murine skin, Piezo1 is upregulated, while knockdown of Piezo1 in dermal fibroblasts attenuates fibrosis [18].

Building on these findings, we hypothesized that Piezo1 in UC-MSCs is regulated by ECM stiffness, and thus contributes to their mechanoresponsive ECM remodeling behavior. To test this, we employed a ruthenium-catalyzed, photo-crosslinked skin-derived ECM hydrogel platform that enables precise control of ECM hydrogel stiffness.

## 2 Materials and methods

### 2.1 Cell isolation and culturing

Human umbilical cords were obtained from the Endothelial Cell Facility at the University Medical Center Groningen (UMCG, Groningen, Netherlands), where they were sourced from anonymous donors in the Department of Gynecology who had provided informed consent for the use of their cord tissue for research purposes. Endothelial Cell Facility is approved under the non-WMO regulation for the use of umbilical cord tissue. Intact cords (>30 cm) were stored at 4 °C in sterile water containing 40 mM KCl (104936, Merck, Darmstadt, Germany), 0.14M NaCl (106404, Merck, Darmstadt, Germany), 11mMD-glucose (G-6152, Sigma-Aldrich, St. Louis, MO, USA), and 10 mM HEPES-Cl (H-7006, Sigma-Aldrich, St. Louis, MO, USA) (pH7.4) for up to four days. In 6 well plates, UC-MSCs were derived by outgrowth culture of rings (2–5 mm, 4 per well) of the cords, after opening the cord and exposing the Wharton's Jelly to the culture substrate. Rings and cells were cultured in UC-MSCs complete culture medium with low-glucose Dulbecco's Modified Eagle Medium (DMEM; BE12-707F, Lonza, Basel, Switzerland), 10% heat-inactivated fetal bovine serum (FBS; Gibco, Sigma-Aldrich, MO, USA), 1% L-glutamine (catalog #17-605E, Lonza BioWhittaker, Verviers, Belgium), and 1% penicillin (#15140122, Gibco Invitrogen, Carlsbad, CA, USA). The medium was refreshed every 3 days. Upon reaching more than 70% confluence, cells were enzymatically dislodged and further cultured in 25 cm^2^ flasks (Passage 1, P1). All cultures were routinely tested for mycoplasma contamination using a PCR screening and confirmed to be mycoplasma-free before use in experiments. After subculturing, P4 cells were used for flow cytometric analyses and differentiation potential evaluation. P5 to P6 generation cells were harvested and used for three-dimensional (3D) cultures in hydrogels.

### 2.2 Flow cytometric analyses

Cultured cells were added at 1 × 10^6^ per tube and collected by centrifugation at 300×*g* at 4 °C, 5min. Next, the cells were resuspended in 100 μL FACS buffer (5 mg/mL bovine serum albumin (BSA; #A9647; Sigma-Aldrich, St. Louis, MO, USA) in phosphate-buffer saline (PBS)) and incubated for 30 min to block non-specific binding. All incubations and washings were done with FACS buffer. Fluorochrome-conjugated primary mouse-anti-human monoclonal antibodies were added to the tubes at a final concentration of 10 μg/mL in 100 μL FACS buffer containing the cells. The following antibodies were used: CD31-PE/Cy (IgG1, #25-0319-41, eBioscience, Thermo Fisher Scientific, Waltham, MA, USA), CD45-FITC (IgG1, #IQP-1, IQ Products, Groningen, Netherlands), CD90-APC (IgG1, #561, BD Pharmingen, Diego, CA, USA), CD34-APC (IgG2a, #130.090–954, Miltenyi Biotec, Bergisch Gladbach, Germany), CD44-FITC (IgG2b, #555478, Pharmingen, San Diego, CA, USA), and CD105-PE/Cy (IgG1, #25-1057-4, eBioscience, Thermo Fisher Scientific, Waltham, MA, USA). Incubation at 4 °C in the dark was for 30min. Isotype control antibodies used were: IgG1-PE/Cy (#25-4714-41, eBioscience, Thermo Fisher Scientific, Waltham, MA, USA), IgG2b-FITC (#555742, BD Pharmingen, San Diego, CA, USA), IgG2a-APC (#550882, BD Pharmingen, San Diego, CA, USA), IgG1-APC (#IQP-191A, IQ Products, Groningen, Netherlands), and IgG1-FITC (#IQP-191F, IQ Products, Groningen, Netherlands). Next, cells were washed twice, resuspended in 300 μL FACS buffer, and analyzed by flow cytometry. Multiparametric flow cytometry was performed on 1 × 10^6^ cells per sample using a BD FACSCanto II system (Becton Dickinson, BD biosciences, Ashland, USA) with BD FACSDiva software. Data analysis was conducted using FlowJo software (FlowJo, LLC, Becton Dickinson, Ashland, USA), with the percentage of positive cells and the mean fluorescence intensity values calculated relative to isotype controls.

### 2.3 UC-MSCs differentiation

The UC-MSCs were seeded at 10,000 cells/cm^2^ in 24-well plates and maintained until reaching 80% confluence. The culture medium was then replaced with differentiation induction media for adipogenic, osteogenic, and myogenic (smooth muscle) differentiation. Inducers were respectively: 0.1 μM dexamethasone (A13449, Gibco, Thermo Fisher Scientific, Waltham, MA, USA), 1 nM insulin delivered via Insulin-Transferrin-Selenium supplement (ITS; 41400-045, Gibco, Thermo Fisher Scientific, Waltham, MA, USA), and 0.5 mM IBMX (3-isobutyl-1-methylxanthine, A0695, AppliChem, Darmstadt, Germany) for Adipocyte induction; 0.1 μM dexamethasone, 10 mM β-glycerophosphate (G9422, Sigma-Aldrich, St. Louis, MO, USA), and 0.05 mM ascorbic acid (LC115309, Fisher Scientific, Fisher Scientific, Hampton, NH, USA) for osteoblasts and 10 ng/mL transforming growth factor beta 1 (TGF-β; 1002110UG, PeproTech, Gibco, Thermo Fisher Scientific, Waltham, MA, USA) for smooth muscle cells. All factors were added to UC-MSCs complete culture medium. Induction media were replaced twice weekly throughout the induction two-week period. Next, cells were washed with PBS and fixed with 2% paraformaldehyde (PFA; Sigma-Aldrich, St. Louis, MO, USA) in PBS for 15min and washed with PBS and stored at 4 °C in PBS until analyses.

Adipogenic differentiation was assessed by Oil Red O staining. A stock solution of 0.5%w/v Oil-Red-O (O0625, Sigma-Aldrich, St. Louis, MO, USA) in isopropanol (190764, Sigma-Aldrich, St. Louis, MO, USA), was diluted with deionized water (dH_2_O) (3:2, v/v) and used to stain the fixed cells for 15min. Upon washing with 60% isopropanol nuclei were stained with hematoxylin for 1 min, followed by rinsing with dH_2_O and slide mounting. Osteogenic differentiation was assessed by Alizarin Red staining. Fixed and washed cells were incubated with 0.5% Alizarin Red (A5533, Sigma-Aldrich, St. Louis, MO, USA) in PBS for 10 min, followed by PBS washing. Nuclei were stained with hematoxylin and rinsed with dH_2_O. For myogenic differentiation fixed cells were permeabilized with 0.25% Triton X-100 (9036-19-5, Sigma-Aldrich, St. Louis, MO, USA) in PBS for 5min, washed with PBS, and incubated with Rabbit anti-Human antibody against alpha-smooth muscle actin (α-SMA; 1:250, 124964, Abcam, Cambridge, UK) for 1h at room temperature. After PBS washing, cells were incubated with Goat anti-Rabbit-HRP conjugate (1:100, P0448, Dako, Glostrup, Denmark) for 30min. Color development was with 3,3′-diaminobenzidine solution (DAB; Dako, Glostrup, Denmark) for 10min after which cells were counterstained with hematoxylin and mounted using glycerol (Sigma-Aldrich, St. Louis, MO, USA) for microscopy.

Imaging of stained cells was done by bright field microscopy (EVOS model M5000, Thermo Fisher Scientific, Waltham, MA, USA).

### 2.4 Generation of ECM hydrogels with different stiffnesses with and without embedded UC-MSCs

Generation of skin-derived decellularized ECM (dECM) hydrogels with varying stiffnesses, was done according to our previously published procedures [19,20]. Briefly, porcine skin (purchased from a slaughterhouse, Kroon Vlees, Groningen, Netherlands) was chopped and blended into a paste, subjected to decellularization and freeze-drying to obtain freeze-dried ECM. This was ground into a powder and dissolved at 2% w/v in 0.01M hydrochloric acid containing 0.2% w/v pepsin (Sigma-Aldrich, St. Louis, MO, USA). After 24h, the homogeneous solution was neutralized to form the dECM pre-gel which was kept at 4 °C.

To prepare the crosslinking solutions, 0.03M and 0.06M pentamethylcyclopentadienyl bis(triphenylphosphine) ruthenium(II) chloride (CAS: 92361-49-4, hereafter referred to as Ru) and 0.1M sodium persulfate (SPS, CAS: 7775-27-1) (both were from a ruthenium visible light photo initiator kit (Advanced BioMatrix, San Diego, California, US) were dissolved in PBS. For the experimental groups, 20 μL of either 0.03M or 0.06M Ru solution and 20 μL of 0.1M SPS solution were added to 1 mL of dECM pre-gel and mixed.

First, cell-free dECM hydrogels were prepared by crosslinking under UV light (20 mW/cm^2^ at 4.5 cm distance using two 9W 405 nm lamps) for 5 min to replicate skin and scar stiffness profiles. The hydrogels were classified based on crosslinking intensity (Ru concentration) as Ru0 (no crosslinking, soft), Ru3 (medium stiffness), and Ru6 (high stiffness).

Then, harvested UC-MSCs were embedded in the dECM pre-gel at a density of 2 × 10^6^ cells/mL. The cell-containing mixture was then plated at 200 μL per well in 48-well plates and incubated at 37 °C for 1h to allow gelation The hydrogels were then crosslinked under the same UV conditions described above. Excess Ru and SPS were removed by washing three times with PBS, and 0.4 mL of UC-MSC complete culture medium was added per well for subsequent culture.

These hydrogels, irrespective of cell embedding, were subjected to analyses at 4h post-crosslinking (referred to as day0, d0), day 1 (d1) and day 5 (d5).

### 2.5 Piezo1 modulation of UC-MSCs in 3D

Piezo1 agonist Yoda1 (SML1558, Sigma Aldrich, Zwijndrecht, Netherlands) was used at 1 μM, (diluted from a 2 mM stock in dimethyl sulfoxide (DMSO; Sigma-Aldrich, St. Louis, MO, USA)). Piezo1 antagonist GsMTx4 (ab141871, Abcam, Cambridge, UK) was used at 5 μM (diluted from a 5 mM stock in PBS). The selected concentrations were based on prior mechanotransduction studies reporting effective and non-toxic Piezo1 activation or inhibition in mesenchymal stem cells and other mechanosensitive cell types, including in both 2D and 3D culture systems [[21], [22], [23], [24]]. Both were added to hydrogels immediately after photocrosslinking and PBS washes. Vehicle controls were respectively DMSO for Yoda1 (Crtl-Y) and PBS for GmsMtx4 (Ctrl-G) at the same concentration as the diluent with the active compounds. This setup resulted in four experimental groups: Ctrl-Y, Yoda1, Ctrl-G and GsMTx4. All treatments were maintained throughout the experiment, with treatments being refreshed each time the medium was replaced (every 2 days).

### 2.6 Mechanical properties measurement

A low load compression tester (LLCT, Mytri Aeldoom, the Netherland) was used to test the viscoelastic properties of hydrogels [25,26]. To assess the physiological relevance of hydrogel stiffness, human abdominal skin was also measured (Skin obtained from patients who underwent surgery at the department of Plastic Surgery in the UMCG. Ethical approval was waived by the Institutional Review Board (Ref. No. M24.332256). The skin sample was obtained as anonymized discarded surgical waste material in accordance with institutional ethical guidelines. LabVIEW 7.2 program (National Instruments, Austin, USA) was used for the LLCT load cell and linear positioning for control and data acquisition. The LLCT was equipped with a 2.5 mm diameter probe, which applied a 20% compression deformation (0.2 strain) to the hydrogels within 1 s (strain rate, $\dot{\varepsilon }$ = 0.2s^−1^) and maintained compression for 100s. Stress (σ), defined as applied force (F) divided by probe area, was plotted as a function of strain and the slope of the stress-strain linear fitting was taken as the stiffness (Pa or N/m^2^). Subsequently, during the 100s period of constant strain, the relaxing stiffness (*E(t)*) was represented as stress (σ(t)) divided by 0.2 strain. A Maxwell function (Eq. (1)) was fitted to *E(t)* curve to reveal how many Maxwell elements (MEs) and their corresponding relaxation time constant (*τ*_*i*_) & relative importance (*R*_*i*_, Eq. (2)) were involved in the stress relaxation process. The minimum number of MEs (n) was determined by monitoring the decrease in chi-square function expressed by Eq. (3) over N measurement points.

(1) $$E\left(t\right)=E_{1}e^{\frac{t}{\tau_{1}}}+E_{2}e^{\frac{t}{\tau_{2}}}+E_{3}e^{\frac{t}{\tau_{3}}}+\cdots +E_{n}e^{\frac{t}{\tau_{n}}}$$

(2) $$R_{i}=\frac{E_{i}}{E_{1}+E_{2}+E_{3}+\ldots {+E}_{n}}$$

(3) $$\chi^{2}=\sum_{j=0}^{100}\left[\frac{E_{j}-E\left(t_{j}\right)}{\sigma_{j}}\right]$$

#### 2.6.1 Volume normalization of stiffness & stress relaxation

During UC-MSC culture, volume reduction of hydrogels was observed, resulting from active cell-mediated contraction in response to the surrounding matrix environment. The volume (*V,* in mm^3^) of the hydrogels was determined at defined time points during the culture period.

The surface area (*A*, in mm^2^) was monitored using a regular flatbed scanner and analyzed with Fiji software [27]. The hydrogels’ thickness (*H*, in mm) was acquired from the LLCT measurements. Hydrogel volume (mm^3^) was calculated using the formula:

(4) $$V\left({mm}^{3}\right)=A\left({mm}^{2}\right)\times H\left(mm\right)$$

To distinguish cell-mediated contraction from cell culturing medium mediated hydrogel deformation, cell-free hydrogels were cultured under identical conditions and analyzed in parallel.

The volume measurements were then used to normalize for contraction-induced increases of matrix density and hence increased stiffness, as well as decreased stress relaxation. This normalization allowed us to separate effects of cell-based matrix remodeling from sheer volume reduction.

Volume-normalized stiffness (*E*_*norm*_) was defined using the formula:

(5) $$E_{norm}\left(N\cdot mm\right)=E_{raw}\left(MPa:\frac{N}{{mm}^{2}}\right)\times V\left({mm}^{3}\right)$$

Volume-normalized stress relaxation (*SR*_*norm*_) was calculated as:

(6) $${SR}_{norm}\left({mm}^{-3}\right)={SR}_{raw}\left(\%\right)÷V\left({mm}^{3}\right)$$

Where *SR*_*norm*_ = volume-normalized stress relaxation; *SR*_*raw*_ = raw stress relaxation; *V* = hydrogel volume.

This normalization assumes an approximately linear scaling of bulk mechanical properties with effective matrix volume (as a proxy for matrix density) over the limited strain range probed in our LLCT measurements (20% compression). We emphasize that this approach represents a phenomenological, first-order approximation, intended to facilitate comparison between conditions and to decouple geometric compaction from intrinsic ECM remodeling, rather than a full constitutive model of the ECM, which is known to exhibit nonlinear, strain-dependent fiber-network mechanics.

### 2.7 Cell viability staining

A total of five donors in two replicate hydrogels per condition were used. Hydrogels were stained with 5 μM calcein AM (C1430, Thermo Fisher Scientific, US) for live cells and 3 μM propidium iodide (PI; P4170, Sigma Aldrich, St. Louis, MO, USA) for dead cells and 8 μM Hoechst (Hoechst 33342, Thermo Fisher Scientific, US) for cell nuclei at day 1 and day 5. Imaging was with an EVOS inverted microscope (EVOS model M5000, Thermo Fisher) with GFP (λex 470/522 nm/λem 525/550 nm), Texas Red (λex 585/629 nm/λem 628/632 nm) and DAPI (λex 335/379 nm/λem 417/477 nm) filters. Per hydrogel, a central image at 10× magnification was randomly selected.

CellProfiler 4.2.1 software was used to process fluorographs [28]. Cell viability was calculated as the fraction of live (green with blue nucleus) over dead (red nucleus) cells. Cellular aspect ratios were derived from each cell's major and minor axis using the fitted ellipse method. The average 2D projected cell area (calcein AM staining) was used as a measure for cell spreading.

### 2.8 ECM histochemical staining

Cell-free hydrogels and cell-embedded hydrogels (d1 and d5) were fixed in 4% PFA in PBS at 37 °C for 30 min. After three PBS washes, the fixed hydrogels were embedded in 2% agarose (Invitrogen, Waltham, MA, USA) in PBS to prevent contraction during dehydration. These samples were embedded in paraffin. Sections (4 μm) were processed for further analyses.

Sections were stained with Weigert's iron hematoxylin (Sigma Aldrich, St Louis, MO, USA) for 10min, washed under running tap water (10min) and subsequent staining with 0.1% picrosirius red (PSR; Sigma-Aldrich, St. Louis, MO, USA) in 1.3% picric acid (Sigma-Aldrich, St. Louis, MO, USA) for 1h. After washing with 0.5% acetic acid and dehydration with 100% ethanol, slides were resin-mounted. Slides were scanned with a Hamamatsu NanoZoomer digital slide scanner at 40× magnification (Hamamatsu Photonic K.K., Japan) and images viewed using Aperio ImageScope V 12.4.6 (Leica biosystem).

Collagen fibers of cell-embedded hydrogels were quantified with CellProfiler 4.2.1 software. For each of the five cell donors, three randomly selected regions of interest (ROI: 2000 x 2,000dpi i.e. 467 μm × 467 μm) were analyzed from one hydrogel section per donor. The fibers' fractional area (FA) was calculated as the fraction of area occupied by stained fibers over the total area of the ROI. The fibers’ mean intensity (MI) staining was calculated by dividing the sum of all PSR-positive pixel intensities by the area occupied by PSR-positive pixels within the ROI. Cells were often surrounded by holes or voids. The area of non-stained cell-surrounding holes was calculated as the fraction of holes over total area of the ROI surrounding cells using Fiji.

#### 2.8.1 Volume-normalized collagen fiber analysis

To account for hydrogel contraction-induced densification, FA (fractional area) was normalized to the hydrogel volume:

(7) $${FA}_{norm}={FA}_{raw}\left(\%\right)\times V\left({mm}^{3}\right)$$

where *FA*_*norm*_ = volume-normalized fractional area; *E*_*raw*_ = raw fractional area; *V* = hydrogel volume (see Section 2.6.1). This correction aimed to eliminate the increase in FA caused by contraction-driven collagen compaction. In contrast, MI did not require normalization because MI is an intensity-based measurement that is independent of hydrogel density.

### 2.9 Immunofluorescence staining

Antigen retrieval was done via heating sections in 10 mM citrate buffer pH6 at 85 °C for 4h. Upon PBS washes, sections were blocked in 4% BSA in PBS for 15min followed by incubation with primary antibody (See Table 1 for details) at room temperature for 1h. After three PBS washes, sections were incubated with secondary antibody (See Table 1 for details) and DAPI (4′,6-diamidino-2-phenylindole, Thermo fisher scientific, Waltham, MA, USA; 1:7500) in 2% BSA in PBS at room temperature for 1h. After PBS washes, sections were mounted in Citifluor mounting medium (Science Services, Munich, Germany). Imaging was done with a Leica SP8 X white light laser confocal microscope (Leica, Wetzlar, Germany), λex 405 nm/λem 455 nm for DAPI and λex 630 nm/λem 665 nm for Piezo1/MMP1/MMP2/MMP9/MMP14 at 40× magnification.

**Table 1:** Details of the antibodies used for immunofluorescence detection in the study.

Antibody Type	Target/Specificity	Host	Fluorophore	Company	Catalog Number	dilution
Primary	Piezo1	Mouse	None	Novus Biologicals	NBP2-75617	1:200
Primary	MMP1	Rabbit	None	Abcam	ab52631	1:100
Primary	MMP2	Rabbit	None	Abcam	ab37150	1:200
Primary	MMP9	Rabbit	None	Thermo Fisher	MA5-15886	1:100
Primary	MMP14	Rabbit	None	Abcam	ab51074	1:200
Secondary	Goat Anti-Mouse	–	Alexa Fluor™ 647	Invitrogen	A21235	1:400
Secondary	Goat Anti-Rabbit	–	Alexa Fluor™ 647	Invitrogen	A21244	1:400

The mean intensities of Piezo1, MMP2, and MMP14 staining were calculated using CellProfiler by dividing the sum of all Piezo1 positive pixel intensities by the area occupied by Piezo1 positive pixels within the ROI. Two areas (1,160μm × 1,160 μm: 4 × 4 tiles) in hydrogel sections with UC-MCSs of three donors were assessed. From two areas, five tiles (290μm × 290 μm) containing at least five nuclei were randomly selected and quantified in detail.

### 2.10 Statistical analyses

All statistical analyses were performed using GraphPad Prism v9.2.0 (GraphPad Company, San Diego, CA, US). All data are shown as mean ± standard deviation from the experiments that were performed with at least three independent experiment replicates, each using cells from different donors. The figure legends indicate the sample size associated with each experiment. A mixed-effects model using restricted maximum likelihood (REML) was employed to account for repeated measures across biological replicates (donors). The hydrogel treatment group was modeled as a fixed effect, and donor was included as a random effect. Post-hoc comparisons were conducted using Tukey's multiple comparisons test. Data populations were considered significantly different at p < 0.05.

## 3 Results

### 3.1 Culture and characterization of UC-MSCs

The MSC nature of the cultured UC-MSCs was confirmed by their spindle-like morphology (Fig. S1A), expression of CD90, CD44 and CD105 while CD45, CD34, and CD31 were absent (Fig. S1B). Furthermore, MSC confirmation was from their differentiation capacity to adipocytes, osteoblasts and smooth muscle cells (Fig. S1C).

### 3.2 The mechanics and shapes of ECM hydrogels are affected by UC-MSCs

#### 3.2.1 The control, mechanically stable cell-free ECM hydrogels replicate skin and scar stiffness profiles

**Fig. 1:**
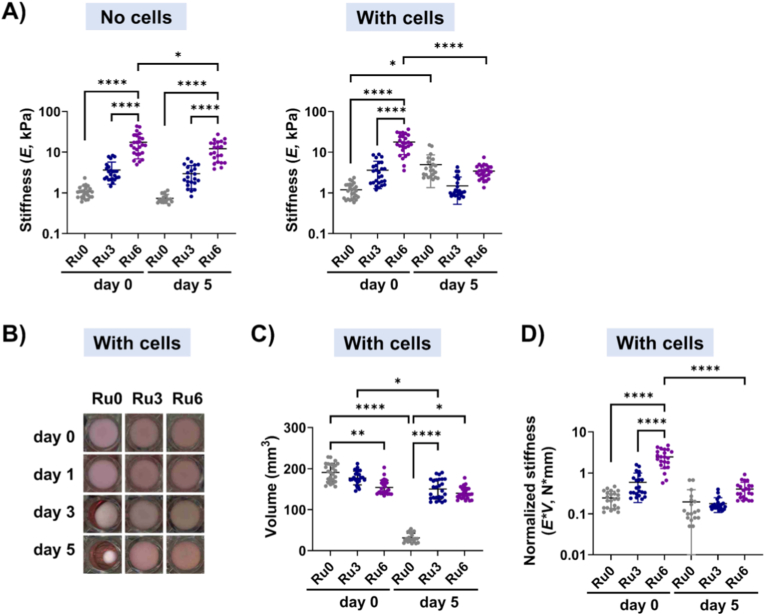
**Hydrogel stiffness, volume, and volume-normalized stiffness of crosslinked hydrogels with/without UC-MSCs.** A) The stiffness (E) of cell-free (left) and UC-MSC-embedded (right) skin ECM hydrogels (Ru0 (grey dots), Ru3 (blue dots), and Ru6 (purple dots)) on d0 and d5; B) Hydrogel shape changes (top view): Representative images of the same hydrogels on days 0, 1, 3 and 5, showing contraction; C) Volume (*V*, mm^3^) of cell-embedded ECM hydrogels on d0 and d5; D) volume-normalized stiffness (*E*_*norm*_ = *E*_*raw*_ × *V* in *N.mm*) of cell-embedded hydrogels on d0 and d5, which decoupled geometric effects (hydrogel contraction) from intrinsic matrix remodeling. Data are presented as mean ± standard deviation, with each dot representing technical measurements (2 independent tests per hydrogel) from two hydrogel replicates for each of the UC-MSC donors (n = 5). Statistical significance was analyzed by a mixed-effects model using restricted maximum likelihood estimation (REML) (*p < 0.05, **p < 0.01, ***p < 0.001, ****p < 0.0001).

Initial stiffnesses (d0), increased in a Ru-concentration-dependent fashion: Ru0 (1.1 ± 0.4 kPa), Ru3 (3.7 ± 2.0 kPa), and Ru6 (17.5 ± 10.7 kPa) (p < 0.0001 for Ru0 vs. Ru6 and Ru3 vs. Ru6; Fig. 1A). The Ru3 hydrogels resembled human skin (2.6 ± 2.2 kPa, Fig. S2) while Ru6 would mimic dermal scars. Compared to skin (75.2 ± 6.6%, Fig. S2), all hydrogels had a higher stress relaxation (Fig. S3A): Ru0 95.6 ± 4.1%, Ru3 80.9 ± 8.3% and Ru6 86.3 ± 7.4% (p < 0.0001 for Ru0 vs Ru3, P = 0.0003 for Ru0 vs Ru6). Except for stiff hydrogels (17.5 ± 10.7 kPa to 12.1 ± 6.6 kPa, d0 to d5, p = 0.0128), cell-free hydrogels’ stiffness did not change over time. Stress relaxation remained similar over time irrespective of crosslinking.

#### 3.2.2 UC-MSCs embedding preserves initial hydrogels’ stiffness and stress relaxation

Initial stiffness values (d0) of the hydrogels with embedded cells were comparable to the cell-free controls: 1.2 ± 0.5 kPa, 3.6 ± 2.3 kPa, and 17.7 ± 8.9 kPa for Ru0, Ru3, and Ru6 respectively (p < 0.0001 for Ru0/Ru3 vs Ru6) (Fig. 1A). All three groups exhibited nearly fully stress-relaxed (>83% reduction after 100 s) under constant strain in a similar fashion (Fig. S3A).

#### 3.2.3 Cells alter the ECM hydrogel stiffness over time in a stiffness-dependent manner

Over 5 days culture (Fig. 1), UC-MSCs altered the mechanical properties of the hydrogels in a stiffness-dependent manner. UC-MSCs increased stiffness of soft (Ru0) hydrogels, from 1.2 ± 0.5 kPa to 4.9 ± 3.6 kPa (p = 0.0253). Medium stiffness (Ru3) hydrogels kept their initial stiffness (3.6 ± 2.3 kPa vs. 1.5 ± 1.0 kPa, NS). In contrast, UC-MSCs reduced stiffness of Ru6 hydrogels from 17.7 ± 8.9 kPa to 3.4 ± 1.3 kPa (p < 0.0001, Fig. 1A). In fact, at d5 initial stiffness differences between Ru0, Ru3 and Ru6 had disappeared with all three hydrogels reaching approximately 1–5 kPa.

#### 3.2.4 Stiffness-dependent volume contraction of UC-MSC-embedded hydrogels

The volume contraction of ECM hydrogels with embedded UC-MSCs was monitored over 5 days (Fig. 1B &C). On d0, stiff (Ru6) hydrogels exhibited a smaller initial volume compared to the controls (Ru0 vs. Ru6: 190.9 ± 17.4 mm^3^ vs. 154.5 ± 17.4 mm^3^, p = 0.0015, Fig. 1C). This initial volume reduction was likely due to the photochemical crosslinking process. After 5 days of culture, both Ru0 and Ru3 hydrogels showed significant contraction compared to their initial volumes (Ru0: from 190.9 ± 17.4 to 31.5 ± 11.3 mm^3^, p < 0.0001; Ru3: from 177.3 ± 16.8 to 150.1 ± 24.5 mm^3^, p = 0.02), whereas the Ru6 hydrogel maintained a similar volume (from 154.5 ± 17.4 to 140.1 ± 14.3 mm^3^). Unlike the initial contraction, these subsequent volume reductions were likely due to cell-mediated remodeling of the hydrogel matrix.

In contrast, cell-free ECM hydrogels (Ru0, Ru3, and Ru6) showed volumes comparable to their cell-embedded counterparts at d0 (Fig. S4 and 181.3 ± 24.2, 175.6 ± 18.9, and 155.5 ± 10.4 mm^3^, respectively) and exhibited no significant volume changes after 5 days of incubation (Fig. S4 and 171.6 ± 18.6, 161.7 ± 13.4, and 145.1 ± 13.2 mm^3^, respectively). These findings confirm that the volume contraction observed in UC-MSC-embedded hydrogels was primarily driven by cell-mediated processes.

#### 3.2.5 Volume-normalized stiffness and stress relaxation: distinguishing stiffness changes driven by cell-mediated ECM intrinsic remodeling from hydrogel contraction

The use of volume-normalized stiffness (*E*_*norm*_) allowed for identification of cell contraction-independent stiffness changes i.e. caused by ECM remodeling. On d0, the *E*_*norm*_ of the hydrogels increased in a stiffness-dependent fashion. Except in the case of Ru6, which differed from both Ru0 and Ru3 (Fig. 1D, *E*_*norm*__Ru0: 0.26 ± 0.11 N mm; *E*_*norm*__Ru3: 0.59 ± 0.40 N mm; *E*_*norm*__Ru6: 2.48 ± 1.19 N mm p < 0.0001 for Ru0 vs Ru6 and Ru3 vs Ru6). After 5 days, *E*_*norm*_ of both Ru0 and Ru3 hydrogels had not changed compared to d0 (Fig. 1D), suggesting that the observed differences in measured stiffness (Fig. 1C) had been caused by gel compaction through cell contraction only. In contrast, stiff (Ru6) hydrogels had undergone extensive ECM remodeling as their *E*_*norm*_ decreased over 5 days of culture (Fig. 1D, d0 vs d5, p < 0.0001).

The stress relaxation of hydrogels exceeded 77% irrespective of crosslinking and culture time. Yet, at d0, cell-free hydrogels without crosslinking (Ru0) relaxed more than crosslinked hydrogels, while at d5 both soft (Ru0) and stiff (Ru6) hydrogels had lost part of their relaxation (Fig. S3A). At d0, hydrogels with embedded UC-MSCs had a similar volume-normalized stress relaxation across soft (Ru0), medium (Ru3), and high stiffness (Ru6) conditions (Fig. S3). In contrast, at d5 the control (Ru0) volume-normalized stress relaxation had increased compared to all other groups (stiffness and culture time).

### 3.3 UC-MSCs induce stiffness-dependent changes in collagen fibers

**Fig. 2:**
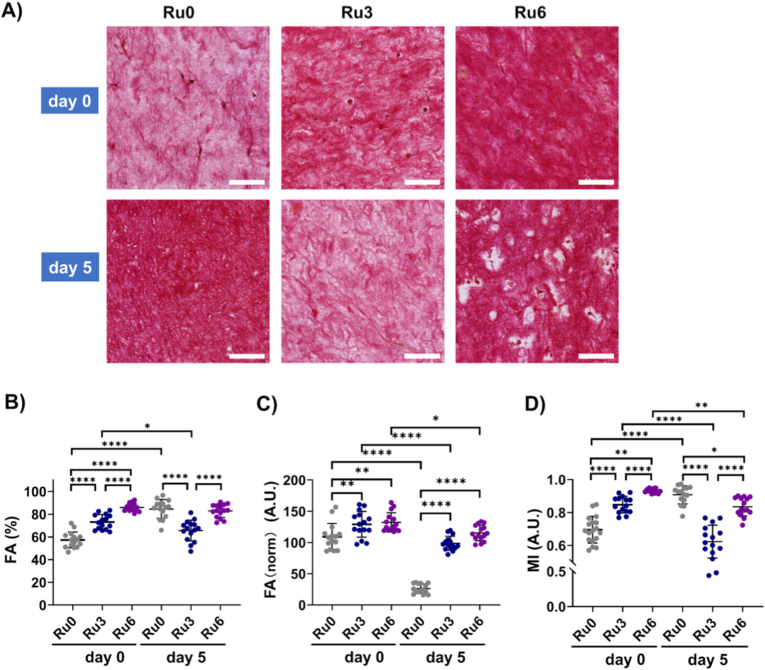
**Characterization of ECM protein fiber structural changes induced by UC-MSCs embedded in ECM hydrogels with variable stiffness.** A) Representative micrographs of PSR staining on skin-derived ECM hydrogels (Ru0, Ru3, Ru6) on d0 and d5 (cell nuclei-blue black; collagen fiber-red). Scale bars: 100 μm. B) fiber fractional area (FA) and C) Hydrogel volume-normalized FA (*FA*_*norm*_ = *FA*_*raw*_ (%) × *V*(mm^−3^)), accounting for contraction. D) mean intensity (MI). PSR: Picrosirius red. FA was calculated by dividing the stained fiber area by the total image area. MI was determined by dividing integrated density (the sum of pixel intensities within the PSR-positive regions) by the total area of the positive pixels, representing the average pixel intensity in the ROI. Each dot represents data from three independent measurements of a single hydrogel per cell donor (n = 5). Statistical significance was analyzed by a mixed-effects model using restricted maximum likelihood estimation (REML) (*p < 0.05, **p < 0.01, ***p < 0.001, ****p < 0.0001).

On d0, irrespective of stiffness, collagen fibers were heterogeneously distributed and randomly arranged, observed as regions with variably dense fiber areas (Fig. 2A). Collagen fiber density increased in a stiffness-dependent fashion as shown by an increased fractional area (FA) and an increased mean intensity (MI) (Fig. 2B). Respective FA values for Ru0, Ru3, and Ru6 hydrogels were 57.4 ± 7.0%, 73.1 ± 6.6%, and 86.0 ± 3.5%, (p < 0.0001 for Ru0 vs Ru3 and Ru0 vs Ru6 and Ru3 vs Ru6). Their corresponding MI values were respectively 0.70 ± 0.08, 0.85 ± 0.05, and 0.93 ± 0.01 (p < 0.0001 for Ru0 vs Ru3 and Ru3 vs Ru6, p = 0.0084 for Ru vs Ru6) (Fig. 2B).

As suggested by the changes in volume-normalized stiffness, embedded UC-MSCs reorganized the collagen fiber architecture during 5d culture (Fig. 2A). Most remarkable was the appearance of pericellular holes or voids in Ru6 hydrogels (Fig. 2A). Except for Ru6 hydrogels, the FA of Ru0 and Ru3 hydrogels changed significantly from d0 to d5: FA increased in Ru0 (57.4 ± 7.0% vs. 84.6 ± 8.2%, p < 0.0001) while it decreased in Ru3 (73.1 ± 6.6% vs. 65.7 ± 9.2%, p = 0.0466). Interestingly, the volume-normalized FA (*FA*_*norm*_) not only differed between stiffnesses in a stiffness-dependent fashion, but also decreased during the 5d culture: on d0, *FA*_*norm*_ were respectively 109.3 ± 21.3, 129.3 ± 20.6 and 132.5 ± 15.4 for Ru0, Ru3 and Ru6 (Ru0 vs. Ru3, p = 0.0091; Ru0 vs. Ru6, p = 0.0014) (Fig. 2C). On d5, *FA*_*norm*_ had decreased to respectively 26.5 ± 7.5, 98.9 ± 11.3 and 115.4 ± 12.4 for Ru0, Ru3 and Ru6 (Ru0 vs. Ru3/Ru6, p < 0.0001). The mean intensities (MI, Fig. 2D) virtually replicated the patterns of FA i.e. a stiffness-dependent increase on d0 which declined on d5 for Ru3 (0.85 ± 0.05 vs. 0.62 ± 0.10, p < 0.0001) and Ru6 (0.93 ± 0.01 vs. 0.84 ± 0.06, p = 0.0016) while MI_Ru0 had increased on d5 compared to d0 (0.70 ± 0.08 vs. 0.91 ± 0.02, p < 0.0001).

To further investigate the influence of long-term culture medium exposure on viscoelastic ECM fiber arrangement, we examined cell-free ECM hydrogels (Ru0, Ru3, and Ru6) incubated for 5 days. At d0, collagen fiber alignment in these acellular hydrogels was comparable to that observed in cell-embedded conditions (Fig. S5). However, after 5 days, all three acellular hydrogels appeared to show a slight reduction in staining intensity upon visual inspection (Fig. S5). This qualitative observation suggests that the viscoelastic nature of the ECM hydrogels may lead to gradual structural relaxation over time, even in the absence of cells actively remodeling the matrix. Such time-dependent relaxation could contribute to the reduction in MI observed in cell-embedded Ru3 hydrogels at d5.

Overall, these findings emphasize the distinct stiffness-dependent role in cell regulated ECM remodeling: in soft Ru0 hydrogels, collagen fibers became denser and more stacked, indicating intrinsic remodeling by cells, while in stiff Ru6 hydrogels, cell-mediated ECM degradation was more prominent.

### 3.4 Sweetspot of spindle formation of UC-MSCs in medium stiffness hydrogels

**Fig. 3:**
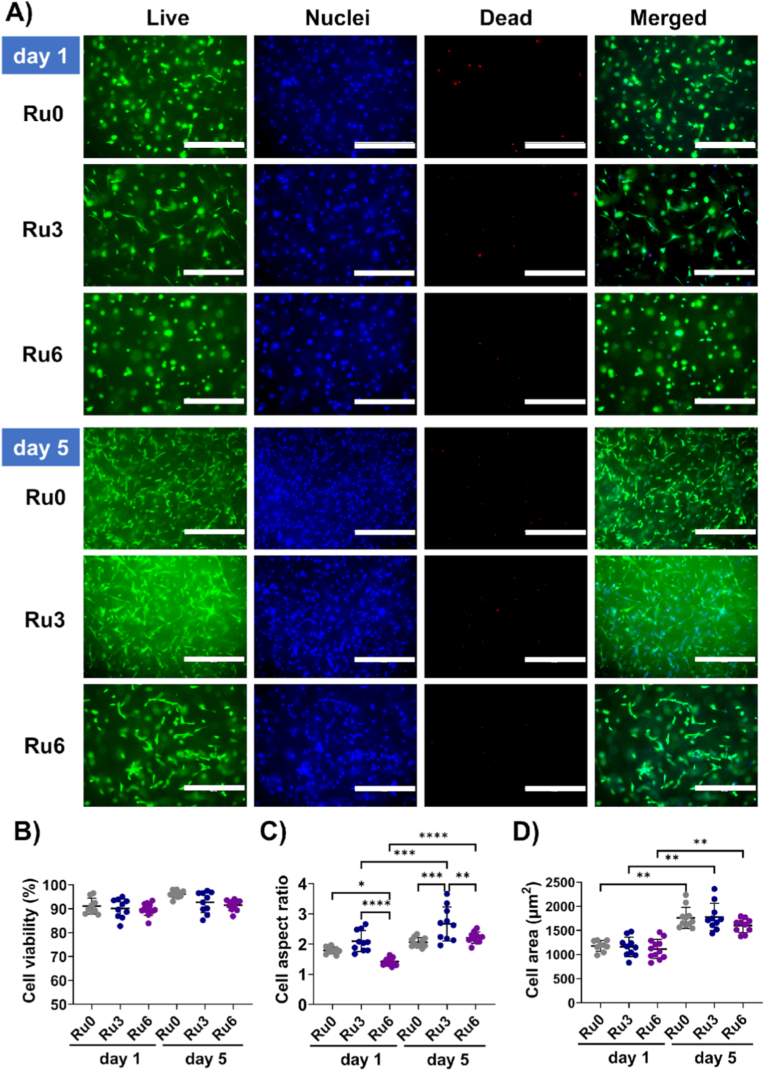
**Morphology and viability of UC-MSCs embedded in ECM hydrogels with variable stiffness over 1 and 5 days.** A) Representative microfluorographs of hydrogel-embedded UC-MSCs (Ru0, Ru3, and Ru6) at d1 and d5. Live cells – green (Calcein AM), nuclei – blue (Hoechst), and dead cells red (PI). B) Quantification of cell viability, expressed as the percentage (%) of live cells (CalceinAM-positive/DAPI-positive). C) Quantification of cell aspect ratio. D) Measurement of the average projected cell area. Cell data were obtained from two replicate hydrogels per cell donor (n = 5 donors). One image per hydrogel was taken at 10× magnification. Scale bars: 400 μm. Statistical significance was analyzed by a mixed-effects model using restricted maximum likelihood estimation (REML) (*p < 0.05, **p < 0.01, ***p < 0.001, ****p < 0.0001).

Neither culture time nor crosslinking affected cells’ viability (Fig. 3). Embedded UC-MSCs spread and acquired a spindle shape between d1 and d5, irrespective of stiffness (Fig. 3A). Yet, spindle formation, i.e. high aspect ratio (width to length), occurred most in medium (Ru3) hydrogels and had started already on d1. Increases were from respectively 1.8 ± 0.1, 2.1 ± 0.3, and 1.4 ± 0.1 for Ru0, Ru3 and Ru6 (P = 0.0134 for Ru0 vs Ru6 and p < 0.0001 for Ru3 vs Ru6) on d1 vs 2.1 ± 0.2, 2.7 ± 0.6, and 2.2 ± 0.2 for Ru0, Ru3 and Ru6 respectively (p < 0.0001 for Ru0 vs Ru3 and p = 0.0024 for Ru3 vs Ru6) on d5 (Fig. 3C). Of note, Ru3 stiffness resembles that of native skin (Fig. S2). The 2D projection of the embedded cells was used as a surrogate measure of spreading (Fig. 3D) which increased over 5d culture from respectively 1.8 ± 0.1, 2.1 ± 0.3, and 1.4 ± 0.1 for Ru0, Ru3 and Ru6 (p = 0.0134 for Ru0 vs Ru6 and p < 0.0001 for Ru3 vs Ru6) to 2.1 ± 0.2, 2.7 ± 0.6, and 2.2 ± 0.2 for Ru0, Ru3 and Ru6 respectively (p < 0.0001 for Ru0 vs Ru3 and p = 0.0024 for Ru3 vs Ru6). Within each stiffness group, spreading increased in time (Ru0: p = 0.0021, Ru3: p = 0.0013, Ru6: p = 0.0050)(Fig. 3D).

### 3.5 The Piezo1 mechanosensor on UC-MSCs responds in a stiffness-dependent fashion

**Fig. 4:**
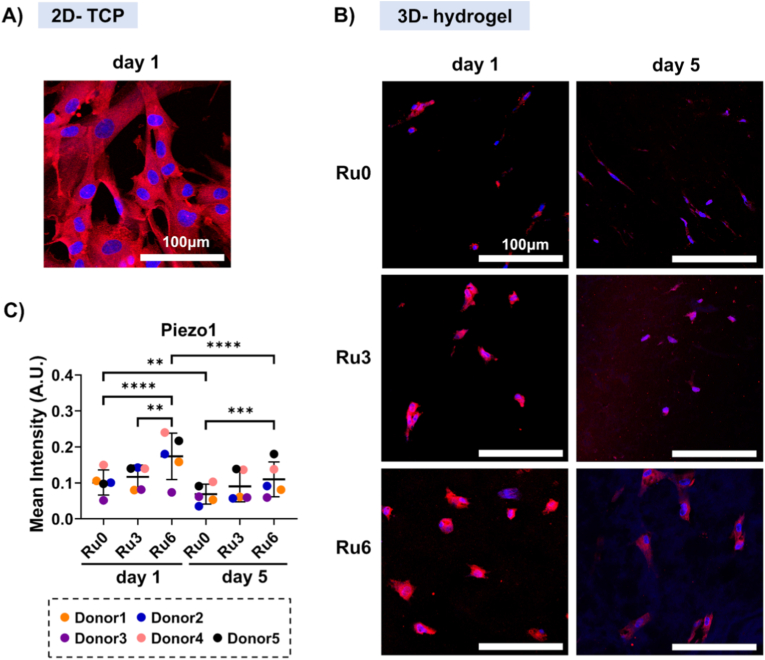
**Stiffness-dependent regulation of Piezo1 in UC-MSCs embedded in ECM.** A) Representative immunofluoro micrographs showing Piezo1 expression (red) in UC-MSCs cultured on TCP plastic (ultrastiff control) and B) in ECM hydrogels (Ru0, Ru3, and Ru6) at d1 and d5. Nuclei: blue (DAPI). C) Relative expression, mean intensity (MI) of Piezo1 by UC-MSCs embedded in ECM hydrogels at d1 and d5. MI was calculated through dividing the total fluorescence pixel intensities by the area occupied by positively stained pixels within the ROI. The data are presented as mean ± standard deviation. Each dot represents the average of five independent tests, performed on random regions of interest within one hydrogel section per donor (n = 5). Statistical significance was analyzed by a mixed-effects model using restricted maximum likelihood estimation (REML) (*p < 0.05, **p < 0.01, ***p < 0.001, ****p < 0.0001).

In controls, i.e. UC-MSCs cultured on (stiff) tissue culture plastic (GPa), Piezo1 (Fig. 4A) and YAP (Fig. S6A) were readily detectable. Embedding in ECM hydrogels, reduced YAP expression but in a time and stiffness-independent fashion (Fig. S6) and was therefore excluded from further study. Similar to YAP, the reduced stiffness of hydrogels compared to plastic, also reduced Piezo1 expression albeit in a time and stiffness-dependent fashion (Fig. 4). At d1, Piezo1 relative expression was respectively 0.10 ± 0.04, 0.12 ± 0.04, and 0.17 ± 0.06 for Ru0, Ru3, and Ru6, (p < 0.0001 for Ru0 vs Ru6, p = 0.0002 for Ru3 vs Ru6, Fig. 4B and C). Piezo1 expression had decreased by d5, showing respectively 0.07 ± 0.03, 0.09 ± 0.05, and 0.11 ± 0.05 for Ru0, Ru3, and Ru6 (p = 0.006 for Ru0, d1 vs. d5; p < 0.0001 for Ru6, d1 vs. d5). Piezo1 remained highest expressed in stiff (Ru6) hydrogels (p = 0.0001 for Ru0 vs. Ru6, Fig. 4C).

### 3.6 Stiffness and contraction of UC-MSCs embedded ECM hydrogels depends on Piezo1 activity

**Fig. 5:**
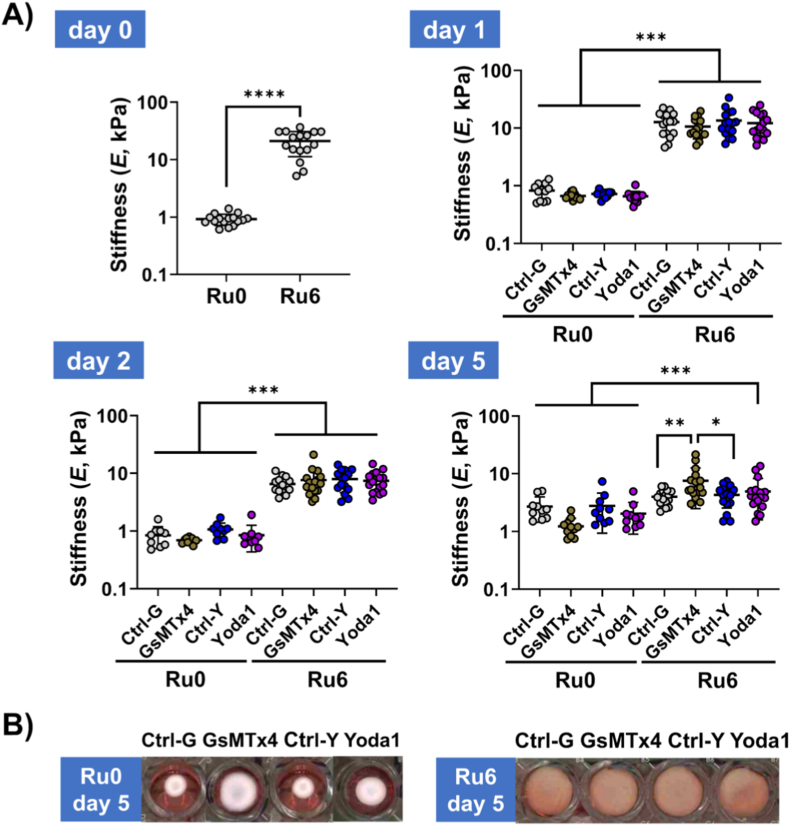
**Effects of Piezo1 modulation on the stiffness and contraction of UC-MSCs embedded ECM hydrogels.** A) Stiffness (*E*) of Ru0 and Ru6 hydrogels before (d0) and under Piezo1 inhibition (GsMTx4) and Piezo1 activation (Yoda1) conditions along with respective controls (Ctrl-G, Ctrl-Y) on d0, 1, 2, and 5. B) Representative images obtained, from a flatbed scanner, of hydrogel shape after d5d of treatment. Data are presented as mean ± standard deviation, with each dot representing technical measurements (2 independent tests per hydrogel) from two hydrogel replicates per cell donor (n = 5). Statistical significance was analyzed by a mixed-effects model using restricted maximum likelihood estimation (REML) (*p < 0.05, **p < 0.01, ***p < 0.001, ****p < 0.0001).

Piezo1 activity was modulated by either agonist Yoda1 or antagonist GsMTx4, with appropriate controls (Ctrl-Y and Ctrl-G). In Ru0 hydrogels (Fig. 5A), compared to untreated hydrogels with embedded cells at day 0 (0.9 ± 0.2 kPa), stiffness remained relatively unchanged at days 1 and 2 across all treatment groups (Ctrl-G, GsMTx4, Ctrl-Y, and Yoda1), with values ranging from 0.8 to 1.0 kPa, indicating the modulation of Piezo1 of hydrogel-embedded UC-MSCs had no influence on the hydrogels’ stiffness. Importantly, treatment with GsMTx4, Yoda1, or their respective controls (PBS and DMSO) did not affect the viability of UC-MSCs in the hydrogels (Fig. S7), confirming that the observed mechanical effects were not due to cytotoxicity.

After 5d, modulation of Piezo1 did not impact on UC-MSCs embedded in Ru0 hydrogels, (Fig. 5A, respectively 2.7 ± 1.2 kPa, 1.2 ± 0.5 kPa, 2.8 ± 1.8 kPa and 2.0 ± 1.1 kPa for Ctrl-G, GsMTx4, Ctrl-Y and Yoda1 treatments). Similarly, in Ru6 hydrogels, stimulation of Piezo1 had no influence in stiffness at d5 (4.3 ± 1.7 kPa for Ctrl-Y vs 4.9 ± 3.3 kPa for Yoda1). However, inhibition of Piezo1 activity (GsMTx4) blocked the expected UC-MSC-induced reduction (Fig. 1) in stiffness at d5 (Figs. 5A and 7.5 ± 5.0 kPa for GsMTx4 vs 4.0 ± 1.2 kPa for Ctrl-G, p = 0.0041). Notably, while in low stiffness hydrogels (Ru0), activation of Piezo1 in UC-MSCs did not influence stiffness, inhibition of Piezo1 had attenuated contraction by d5 (Fig. 5B & Fig. S8A, 32.6 ± 16.8 mm^3^ vs 82.4 ± 13.5 mm^3^ for Ctrl-G vs GsMTx4, p < 0.0001).

In addition, volume-normalized stiffnesses (*E*_*norm*_) followed a similar pattern in which *E*_*norm*_ was comparable in Ru0 hydrogels irrespective of treatment (Fig. S8B, respectively 0.085 ± 0.029, 0.095 ± 0.026,0.089 ± 0.048, and 0.089 ± 0.028 for Ctrl-G, GsMTx4, Ctrl-Y and Yoda1). Yet, in stiff hydrogels (Ru6), inhibition of Piezo1 activity in UC-MSCs had abolished stiffness reduction (Fig. S8B, 0.429 ± 0.118 vs 0.868 ± 0.519 for Ctrl-G vs GsMTx4, p = 0.0002), while stimulation of Piezo1 activity had no effect (0.469 ± 0.218 vs 0.515 ± 0.341 for Ctrl-Y vs Yoda1).

### 3.7 Collagen matrix remodeling by UC-MSCs depends on Piezo1 activity

**Fig. 6:**
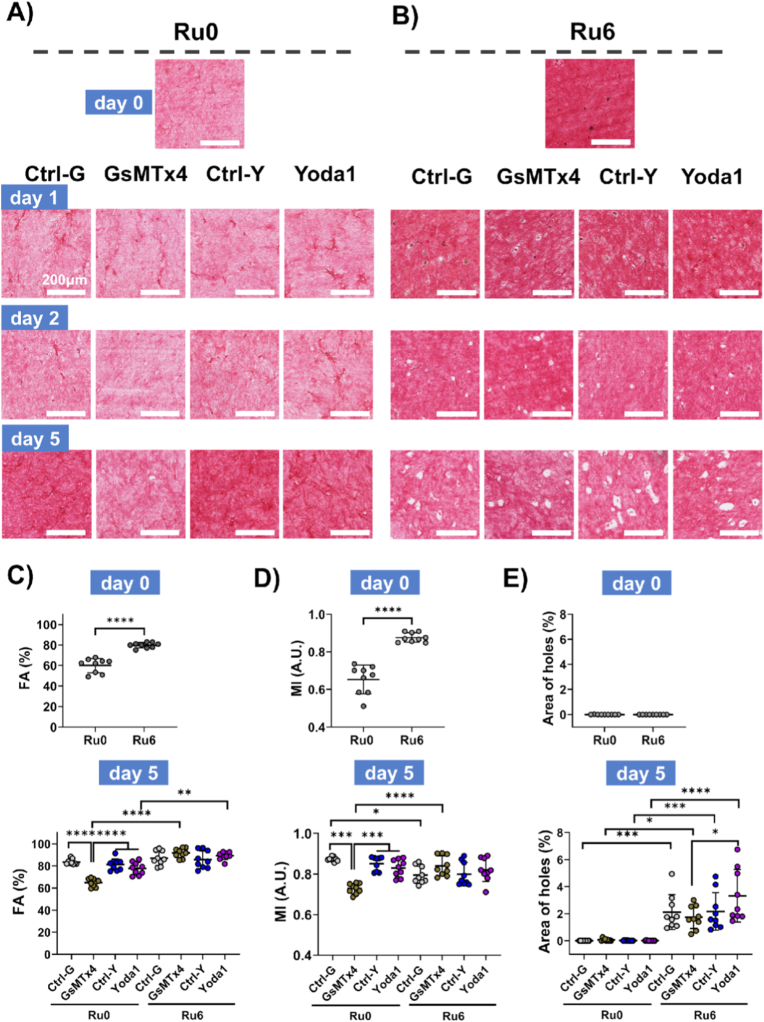
**Collagen matrix remodeling in UC-MSCs embedded ECM hydrogels after Piezo1 modulation.** Representative PSR-stained collagen fibers images for A) Ru0 and B) Ru6 group hydrogels under Piezo1 modulation (Ctrl-G, GsMTx4(inhibition), Ctrl-Y and Yoda1(activation)) at 0, 1, 2, and 5 days of culture (cell nuclei-blue black; collagen fiber-red). Scale bars:100 μm. Quantitative analysis of C) Fractional area (FA), calculated as the stained fiber area divided by total image area; D) Mean intensity (MI), calculated as color intensity divided by the total stained fiber area; and E) Area percentage of collagen holes surrounding cells. Each dot represents data from three independent measurements of a single hydrogel for each cell donor (n = 3). Statistical significance was analyzed by a mixed-effects model using restricted maximum likelihood estimation (REML) (*p < 0.05, **p < 0.01, ***p < 0.001, ****p < 0.0001). PSR: Picrosirius red.

In the soft controls (Ru0, Fig. 6A), embedded UC-MSCs increased the collagen network density over the 5d culture period (Fig. 6A, Ctrl-G, Ctrl-Y). Similarly, activation of Piezo1 had no influence (Fig. 6A, Yoda1). In contrast, inhibition of Piezo1, abolished the collagen remodeling by UC-MSCs (Fig. 6A, GsMTx4). Due to the photocrosslinking, Ru6 hydrogels had a higher collagen density i.e. increased FA and MI than non-crosslinked (Ru0) hydrogels at d0 (Fig. 6A, B, C and D, FA: 60.0 ± 7.0 vs 79.8 ± 2.6, p < 0.0001 and MI: 0.65 ± 0.08 vs 0.88 ± 0.02, p < 0.0001 for Ru0 vs Ru6). As expected (Fig. 2), the collagen density (Fig. 6B) had decreased in control stiff Ru6 hydrogels (Ctrl-G and Ctrl-Y), while stimulation of Yoda1 had no influence (Fig. 6B). At d5 both FA and MI were reduced upon inhibition of Piezo1 in soft Ru0 hydrogels, while in stiff hydrogels, both FA and MI were unaffected by Piezo1 modulation. In detail; for soft Ru0 hydrogels, FA reduction after Piezo1 inhibition vs control: 65.0 ± 3.6 (GsMTx5) vs. 83.5 ± 2.4 (Ctrl-G), p < 0.0001 (Fig. 6C) at d5. While, MI reduction after Piezo1 inhibition for Ru6 was consistent throughout culture, respectively GsMTx4 vs Ctrl-G; d1: 0.60 ± 0.07 vs. 0.67 ± 0.03, p = 0.0318; d2: 0.68 ± 0.03 vs. 0.75 ± 0.05, p = 0.0096; d5: 0.73 ± 0.02 vs. 0.87 ± 0.01, p < 0.0001 (Fig. 6D and Fig. S9).

The matrix remodeling by UC-MSCs of only the stiff hydrogels (Fig. 6B) showed the appearance of the earlier mentioned pericellular holes. Already visible at d2, these holes had become larger by d5 (Fig. 6B–E). However, Piezo1 modulation had no influence on the process of hole formation (Fig. 6E), because neither inhibition nor stimulation differed from their respective controls (Fig. 6E).

### 3.8 Screening of MMP expression in UC-MSCs embedded ECM hydrogels

To assess the involvement of matrix metalloproteinases (MMPs) in stiffness-dependent ECM remodeling, protein expression of MMP1, MMP2, MMP9, and MMP14 was examined in UC-MSCs embedded Ru0 and Ru6 ECM hydrogels at early (day 1) and later (day 5) time points.

At day 1, quantitative immunofluorescence analysis revealed higher expression levels of both MMP2 and MMP14 in stiff Ru6 hydrogels compared to soft Ru0 hydrogels under control conditions (Fig. S10A–D). Specifically, MMP2 expression was increased in Ru6 relative to Ru0 (Ctrl-G Ru0 vs. Ru6: 0.20 ± 0.09 vs. 0.32 ± 0.07, p < 0.0001; Fig. S10B), and a similar stiffness-dependent increase was observed for MMP14 (Ctrl-G Ru0 vs. Ru6: 0.22 ± 0.04 vs. 0.32 ± 0.05, p = 0.0004; Fig. S10D). At day 5, MMP2 expression remained higher in Ru6 hydrogels compared to Ru0 (Ru0 vs. Ru6: 0.19 ± 0.05 vs. 0.27 ± 0.08, p = 0.0003; Fig. S10B). In contrast, MMP14 expression no longer differed between Ru0 and Ru6 hydrogels at this later time point (Ru0 vs. Ru6: 0.19 ± 0.07 vs. 0.23 ± 0.06, n.s.; Fig. S10D). MMP1 and MMP9 exhibited very low immunofluorescence signal intensity across all stiffness conditions at both day 1 and day 5 (Fig. S10E–F). Due to the low signal intensity, reliable quantitative analysis was not feasible, and representative images are sh own.

The effect of Piezo1 inhibition on MMP expression was further evaluated. At day 1, treatment with the Piezo1 inhibitor GsMTx4 did not significantly alter MMP2 expression in either Ru0 or Ru6 hydrogels (Fig. S10B). At day 5, however, MMP2 expression in Ru6 hydrogels was modestly reduced following Piezo1 inhibition (Ru6 Ctrl-G vs. GsMTx4: 0.27 ± 0.07 vs. 0.23 ± 0.08, p = 0.02; Fig. S10B). In contrast, MMP14 expression was not significantly affected by Piezo1 inhibition at either time point or stiffness condition (Fig. S10D).

## 4 Discussion

Mechanotransduction, the process by which cells sense and respond to mechanical cues in their environment, is fundamental to cellular behavior and tissue remodeling. This study examined the stiffness-dependent behavior of UC-MSCs in 3D skin-derived ECM hydrogels and revealed the mechanotransduction signaling involved in mediating these processes. By employing ruthenium-catalyzed crosslinking to modulate skin-derived ECM hydrogels (Ru0, Ru3, and Ru6), we modulated the stiffness of skin-derived ECM hydrogels (Ru0, Ru3, and Ru6). This approach generated a stiffness range from 1.2 ± 0.5 kPa (Ru0) to 17.7 ± 8.9 kPa (Ru6), spanning values representative of normal skin to dermal scar tissue while they all exhibited rapid stress relaxation. After cell-embedding, by decoupling cell-mediated hydrogel contraction from ECM turnover, we demonstrate that UC-MSCs respond to matrix stiffness in a Piezo1-dependent fashion. Specifically, UC-MSCs in low stiffness hydrogel drives contraction while cells in high stiffness hydrogel drives matrix remodeling both as part of the cells' mechanisms to reach an (optimal) intermediate stiffness (1–5 kPa). Furthermore, we identified Piezo1 as a mechanosensor regulating these processes.

Cells exert traction forces on their surrounding matrix, and the resulting feedback between cell-generated forces and matrix resistance defines their morphology and behavior [[29], [30], [31]]. Softer ECMs offer minimal resistance, leading to pronounced contraction [32,33], while stiffer hydrogels provide more resistance, limiting contractibility [19]. Beyond contraction, cellular activity also drives local ECM turnover i.e. molecular ECM rearrangement, deposition and degradation, impacting hydrogel properties [34,35]. To isolate and elucidate the effects of cell induced ECM remodeling and mechanotransduction across hydrogels of varying stiffness, we applied volume normalization of stiffness, stress relaxation, and fiber density. A linear model was assumed, where if a particular property would increase due to contraction, that property was multiplied with the hydrogel volume and vice versa a property which would decrease was divided by hydrogel volume. This normalization rests on the simplified assumption that stiffness scales approximately linearly with hydrogel volume, which serves as a proxy for matrix density. Native and reconstituted ECMs are known to display nonlinear, strain-dependent mechanics arising from changes in their fibrous architecture, and these features are not captured by this linear normalization. For this reason, E_norm_ should be interpreted as a semi-quantitative index that helps to separate contraction-driven densification from intrinsic ECM turnover, rather than as an absolute, model-independent modulus. Notably, the central observation that only the stiff Ru6 hydrogels undergo substantial intrinsic softening, whereas Ru0 changes are dominated by contraction, is already evident from the combination of raw stiffness measurements, volume changes and collagen histology, and is therefore robust to the exact form of the normalization.

UC-MSC morphology and ECM remodeling were significantly influenced by hydrogel stiffness, with cells adopting a more elongated and spindle-like shape in medium-stiffness hydrogels compared to soft and high-stiffness hydrogels. Volume-normalized data further elucidated the role of stiffness and hydrogel contraction in guiding these cellular behaviors and matrix remodeling patterns: In soft hydrogels, the compliance of the matrix resulted in relatively low resistance to cellular traction forces. While mechanosensing remained active, the mechanical feedback received by the cells is diminished compared to stiffer matrices. Consequently, cells contracted the matrix predominantly through pulling ECM fibers closer together to form dense, compact networks and volume reduction, leading to increased stiffness. Medium-stiffness hydrogels appeared to provide an optimal mechanical balance between traction forces and matrix compliance, resembling the stiffness of human abdominal skin. In this environment, UC-MSCs experience sufficient counterforces to sustain traction without excessive dissipation or inhibition. This mechanical equilibrium appears to support a homeostatic cell–matrix interaction, allowing cells to maintain elongated morphologies while minimizing unnecessary remodeling activity. It suggests that medium-stiffness matrices offer the ideal, i.e. homeostatic mechanical environment for cellular mechanosensing (and homeostatic) ECM remodeling. In high-stiffness hydrogels, the matrix exhibits low compliance and resists deformation, thereby limiting cell elongation and spreading. Under these conditions, UC-MSCs experience elevated mechanical resistance upon generating traction forces, which may shift their mechanotransductive response toward matrix remodeling. Rather than relying solely on shape adaptation, cells may initiate ECM degradation and create localized porosity to reduce mechanical constraints. This dynamic, stiffness-dependent behavior not only shows how UC-MSCs adapt their morphology in response to the surrounding matrix, but how these actively remodel the ECM to reach a homeostatic equilibrium. Similar biphasic relationships between stiffness and MSC morphology or differentiation have been reported in other different stiffness matrix systems, supporting the notion that MSCs are finely tuned to their physiological mechanical microenvironment [36,37].

Cells are equipped with multiple mechanotransduction pathways that transmit mechanical cues from the extracellular matrix into intracellular biochemical and biophysical responses. Classical mechanosensing mechanisms rely on integrin-based adhesions, where mechanical resistance is conveyed through focal adhesion complexes and the actomyosin cytoskeleton, enabling force transmission from the ECM toward the cell interior and nucleus. Through these pathways, mechanical inputs regulate cytoskeletal organization, intracellular tension, and downstream signaling or transcriptional responses [38,39]. In addition to these adhesion- and cytoskeleton-mediated pathways, mechanosensitive ion channels such as Piezo1 represent another class of mechanotransducers [40,41]. Piezo1 has been implicated in stiffness sensing across multiple cell types [18,42,43]. Importantly, however, Piezo1 does not directly sense substrate stiffness itself. Instead, structural and biophysical studies have demonstrated that Piezo1 gating is governed by changes in plasma membrane tension and deformation [44]. Mechanical cues originating from matrix stiffness are therefore relayed to Piezo1 indirectly, through stiffness-dependent modulation of cell–matrix adhesions, cytoskeletal prestress, and force transmission to the plasma membrane. Consistent with this mechanistic framework, our findings show that Piezo1 expression in UC-MSCs increases with matrix stiffness. A study by Sun et al. [43] similarly demonstrated that compared to substrates with a stiffness of 62–68 kPa, UC-MSCs cultured on softer matrices (13–16 kPa) exhibited lower Piezo1 levels. This stiffness-dependent expression likely reflects the increased membrane tension and cytoskeletal prestress experienced by cells in stiffer environments. Moreover, differences between 2D and 3D culture systems may further modulate this response. In 3D matrices, cells experience isotropic confinement and homogeneously distributed mechanical cues, which alter the spatial distribution of membrane tension compared to 2D substrates [45]. In fact, on 2D substrates cells are more or less polarized with virtually no mechanical force on their apical, medium-facing, side and most of the mechanical force on their basal, substrate-bound, side. Thus, the stiffness-dependent Piezo1 response observed in our 3D hydrogels emphasizes the importance of three-dimensional matrix context in regulating mechanotransduction.

Importantly, Piezo1 inhibition attenuated UC-MSCs-driven hydrogel remodeling (the softening of stiff matrix through intrinsic ECM remodeling, and the stiffening of soft matrix via contraction), emphasizing its dual role in mechanosensing and matrix remodeling. This finding suggests that Piezo1-mediated calcium influx may act upstream of cytoskeletal tension and ECM turnover pathways, integrating external mechanical signals with intracellular contractility and remodeling machinery. The loss of stiffness adaptation upon Piezo1 inhibition supports a model in which Piezo1 serves as a feedback regulator: when cells experience suboptimal mechanical environments, Piezo1 signaling directs cytoskeletal reorganization and ECM remodeling to restore equilibrium stiffness.

Mechanistically, Piezo1 activation couples membrane tension to actomyosin contractility, focal adhesion dynamics, and force transmission at the cell–matrix interface. Increased calcium influx through activated Piezo1 activates downstream effectors that regulate cytoskeletal organization and adhesion complex turnover, thereby shaping how cells generate and adapt traction forces within three-dimensional matrices. In pathological contexts, such Piezo1-dependent mechanoadaptive signaling has been directly linked to ECM remodeling. For example, Piezo1 activation promotes mechanically induced cartilage degradation through a YAP–MMP13/ADAMTS5 signaling axis in temporomandibular joint osteoarthritis, directly coupling mechanosensing and mechanotransduction to matrix catabolism [46]. In fibrotic diseases such as in experimental kidney fibrosis, inhibition of Piezo1 with GsMTx4 attenuates fibrosis progression. In vitro, Piezo1 activation induces calcium-dependent calpain-2 signaling in Human Kidney-2 cells, leading to talin-1 cleavage, integrin β1 upregulation, and reinforcement of adhesion-mediated force transmission, thereby promoting profibrotic ECM remodeling [17]. Related mechanisms have also been reported in invasive tumor cells, where stiffness-activated Piezo1 localizes to focal adhesions and enhances integrin–FAK signaling, creating a feedforward loop between cellular contractility, ECM remodeling, and tissue stiffening [47]. Collectively, these studies support the concept that Piezo1 does not merely sense mechanical cues, but actively modulates cytoskeletal tension and adhesion signaling pathways that govern matrix remodeling outcomes.

In our UC-MSC system, imaging and the acquired mechanical data indicated that UC-MSCs in high stiffness hydrogels initiate localized ECM degradation and pore formation, consistent with proteolytic matrix remodeling rather than pure mechanical deformation. To further explore potential molecular contributors, we assessed the protein expression of matrix metalloproteinases MMP1, MMP2, MMP9, and MMP14 (Section 3.8; Fig. S10). Among these, MMP2 and MMP14 showed stiffness-dependent expression, whereas MMP1 and MMP9 were detected at very low levels across all conditions. However, Piezo1 inhibition had only limited effects on MMP expression, with no effect at early time points and only a modest reduction of MMP2 expression in stiff hydrogels at day 5. It is important to note that MMP enzymatic activity was not directly quantified in this study, because commercially available *in situ* MMP activity assays have poor reliability and poor reproducibility unfortunately [4]. Therefore, the interpretation that pericellular void formation and matrix softening reflect protease-mediated ECM degradation should be regarded as a supported interpretation based on combined structural, mechanical, and molecular observations, rather than direct evidence of MMP activity. This interpretation is nevertheless consistent with prior studies showing that MSCs can engage MMP-dependent pericellular matrix remodeling/turnover. In particular, MMP-2 and MT1-MMP (MMP14) have been shown to support bone marrow-derived human MSC invasion through reconstituted human basement membranes by enabling matrix remodeling [48]. Previous works from our group have demonstrated that MMP1 and MMP2 direct MSC-driven matrix turnover [4,5]. Moreover, UC-MSC-conditioned medium attenuates fibrosis by enhancing MMP1 expression and reducing collagen content [6], which again links MSC (secretome) activity to ECM modulation.

Taken together, these observations suggest that Piezo1 may influence ECM remodeling indirectly, by modulating cell behavior in response to mechanical context, rather than by directly controlling the expression of specific matrix-degrading enzymes. In this conceptual view, Piezo1-dependent mechanosensing could bias UC-MSCs toward distinct remodeling strategies—such as matrix compaction or localized remodeling—depending on the surrounding stiffness environment. Protease-mediated ECM turnover may therefore represent a secondary and context-dependent component of the remodeling process, rather than a direct downstream target of Piezo1 signaling. Future studies employing activity-based protease assays, secretome profiling, lineage-specific differentiation and targeted pathway perturbations will be required to delineate the precise molecular mediators linking Piezo1 signaling to ECM remodeling. In parallel, the use of alternative three-dimensional culture formats that improve optical and membrane accessibility, such as thin-layer or engineered micro-3D systems, as well as tension- or Ca^2+^- sensitive biosensors, will be essential for enabling real-time functional interrogation of Piezo1 activity under defined mechanical conditions.

The stiffness-dependent mechanoadaptive behavior of UC-MSCs observed here has important implications for regenerative medicine. In a wound healing context, the ability of UC-MSCs to modulate ECM stiffness through Piezo-1-mediated contraction and remodeling could contribute to scar attenuation and restoration of normal tissue mechanics. From a biomaterial's perspective, our data highlight that medium-stiffness matrices approximating the mechanics of native skin provide a favorable environment for MSC mechanosensing and remodeling. Designing scaffolds with tunable viscoelastic properties within this range could thus enhance MSC-mediated tissue repair outcomes. In summary, this study provides a mechanistic link between matrix stiffness, Piezo1-mediated mechanotransduction, and the remodeling capacity of UC-MSCs. These insights have potential implications for the design of biomaterials and regenerative therapies that harness or direct MSC-mediated ECM remodeling.

## 5 Conclusion

In summary, our study identifies Piezo1 as a central mechanosensor guiding stiffness-dependent ECM remodeling by UC-MSCs in a 3D ECM hydrogel environment. Through Piezo1-depemdent mechanosensing, UC-MSCs contract soft matrices and localized remodel ECM in stiff hydrogels, converging mechanical properties toward a common intermediate stiffness. These insights emphasize the dynamic interplay between cellular traction forces, ECM remodeling mechanisms, and mechanical microenvironments. Understanding this crosstalk may provide valuable guidance for designing engineered tissues and therapeutic strategies targeting fibrosis, wound healing, and regenerative medicine.

## CRediT authorship contribution statement

**Fenghua Zhao:** Conceptualization, Data curation, Formal analysis, Investigation, Methodology, Visualization, Writing – original draft, Writing – review & editing. **Xue Zhang:** Conceptualization, Data curation, Formal analysis, Investigation, Methodology, Visualization, Writing – review & editing. **Theo Borghuis:** Formal analysis, Investigation, Methodology, Writing – review & editing. **Linda A. Brouwer:** Formal analysis, Investigation, Methodology, Writing – review & editing. **Janette K. Burgess:** Conceptualization, Funding acquisition, Supervision, Validation, Writing – review & editing. **Prashant K. Sharma:** Conceptualization, Funding acquisition, Supervision, Validation, Writing – review & editing. **Martin C. Harmsen:** Conceptualization, Funding acquisition, Supervision, Writing – review & editing.

## Declaration of competing interest

The authors declare that they have no known competing financial interests or personal relationships that could have appeared to influence the work reported in this paper.

## Data Availability

Data will be made available on request.
