# Supplementary material for: Piezo1 regulates remodeling of skin-derived extracellular matrix by embedded umbilical cord mesenchymal stem cells in a stiffness-dependent fashion
Source: Mater Today Bio. 2026 Jan 31;37:102883. doi: 10.1016/j.mtbio.2026.102883 (PMC12906116; doi:10.1016/j.mtbio.2026.102883)
Supplement: Multimedia component 1 [file mmc1.docx]

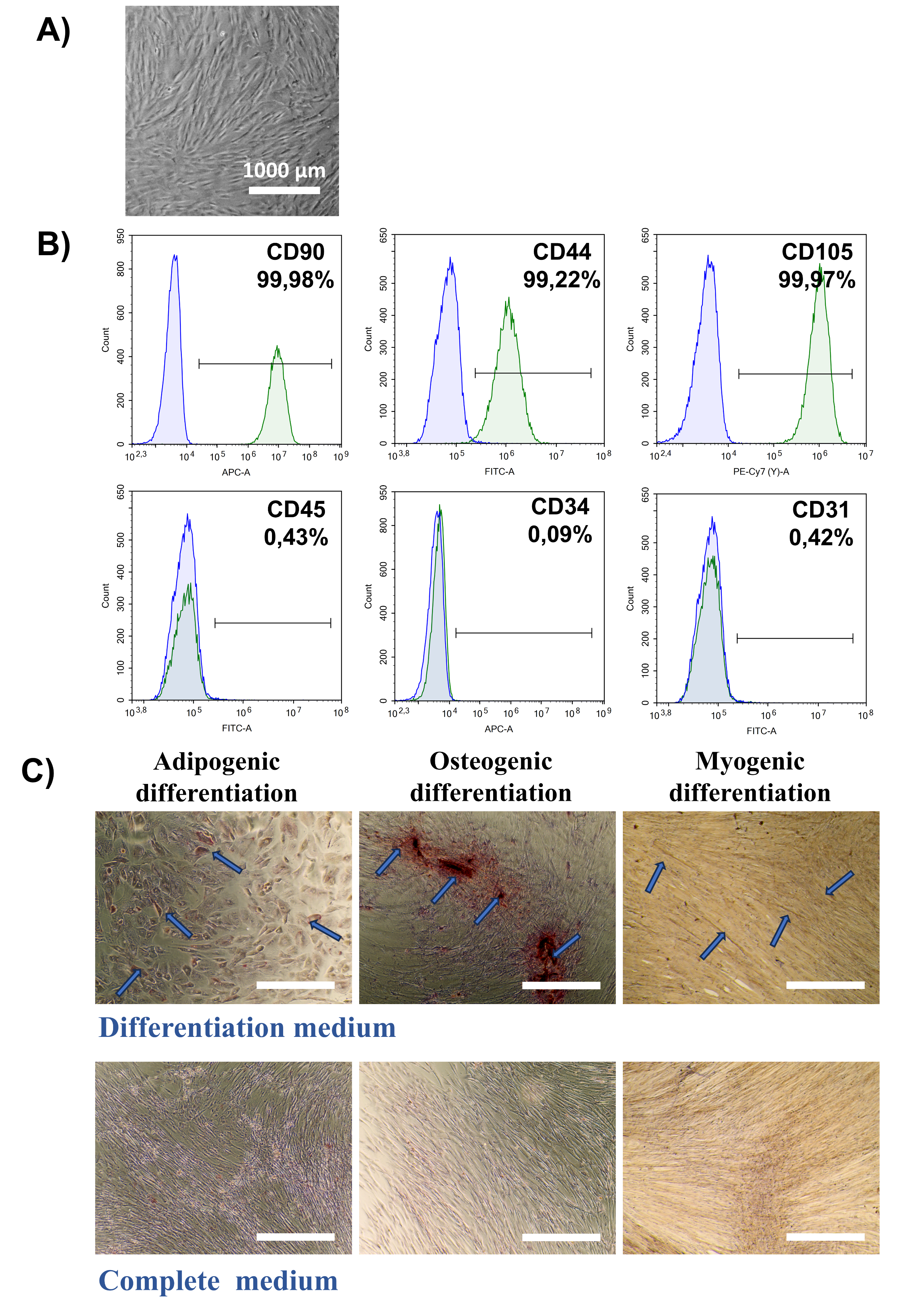


**Fig. S1. Characterization of cultured UC-MSCs.** A) Cell morphology of UC-MSCs on reaching 80% confluence. Scale bar = 1000 µm. B) FACS analysis of UC-MSCs for the surface markers CD31, CD34, CD45, CD44, CD90, and CD105. UC-MSCs were negative for hematopoietic/endothelial markers (CD31, CD34, CD45; <1% positivity) and positive for MSC markers (CD44, CD90, CD105; >99% positivity (n=5)). Histograms show isotype controls (purple) versus antigen-specific staining (green). C) Differentiation potential identification of UC-MSCs after 2 weeks treatment with complete medium and differentiation induction medias. Adipogenesis as detected by the formation of lipid vacuoles, stainable with Oil Red O. Osteogenesis as detected by the formation of calcium deposits, stainable with Alizarin Red S. Myogenic differentiation, specifically toward SMCs, was confirmed by α-SMA expression through immunohistochemistry staining. Blue arrows highlight positively stained regions. Scale bar = 1000 µm. Data show representative images from five independent donors.

Abbreviations: UC-MSC, umbilical cord mesenchymal stem cells; FACS, Fluorescence-Activated Cell Sorting (a specialized type of flow cytometry); SMCs, smooth muscle cells; α-SMA, α-smooth muscle actin.

**A) B)**


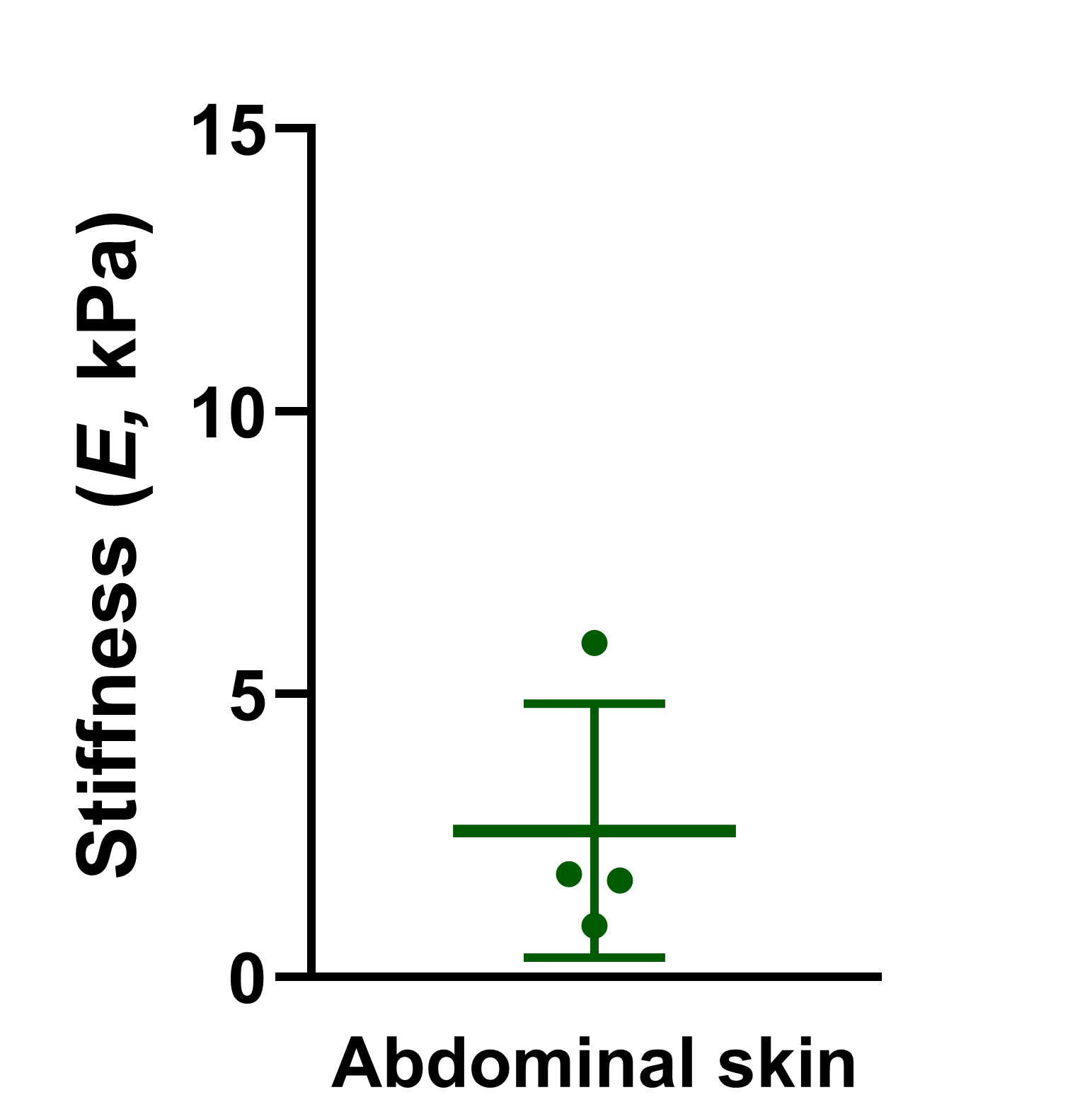

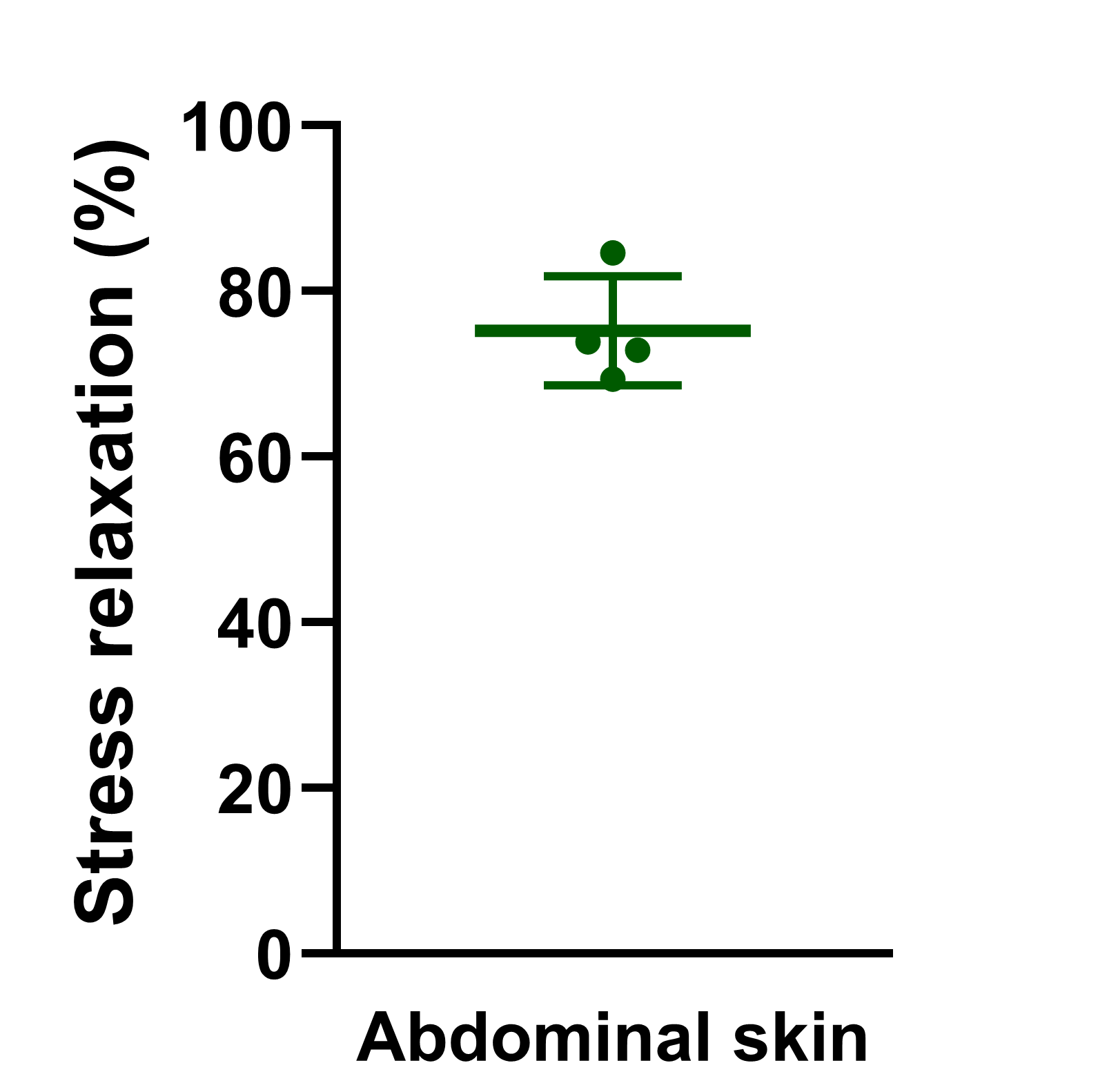


**Fig. S2.** The mechanical properties of the normal human abdominal skin tissue. A) Stiffness; B) Stress relaxation. The data represents the mean ± standard deviation generated from four independent experiments on one piece of skin sample obtained from a 25-year-old patient (gender not available) undergoing plastic surgery at the Department of ODBC (Operatief DagBehandel Centrum), University Medical Center Groningen (UMCG). Ethical approval was waived by the Institutional Review Board (Ref. No. M24.332256).


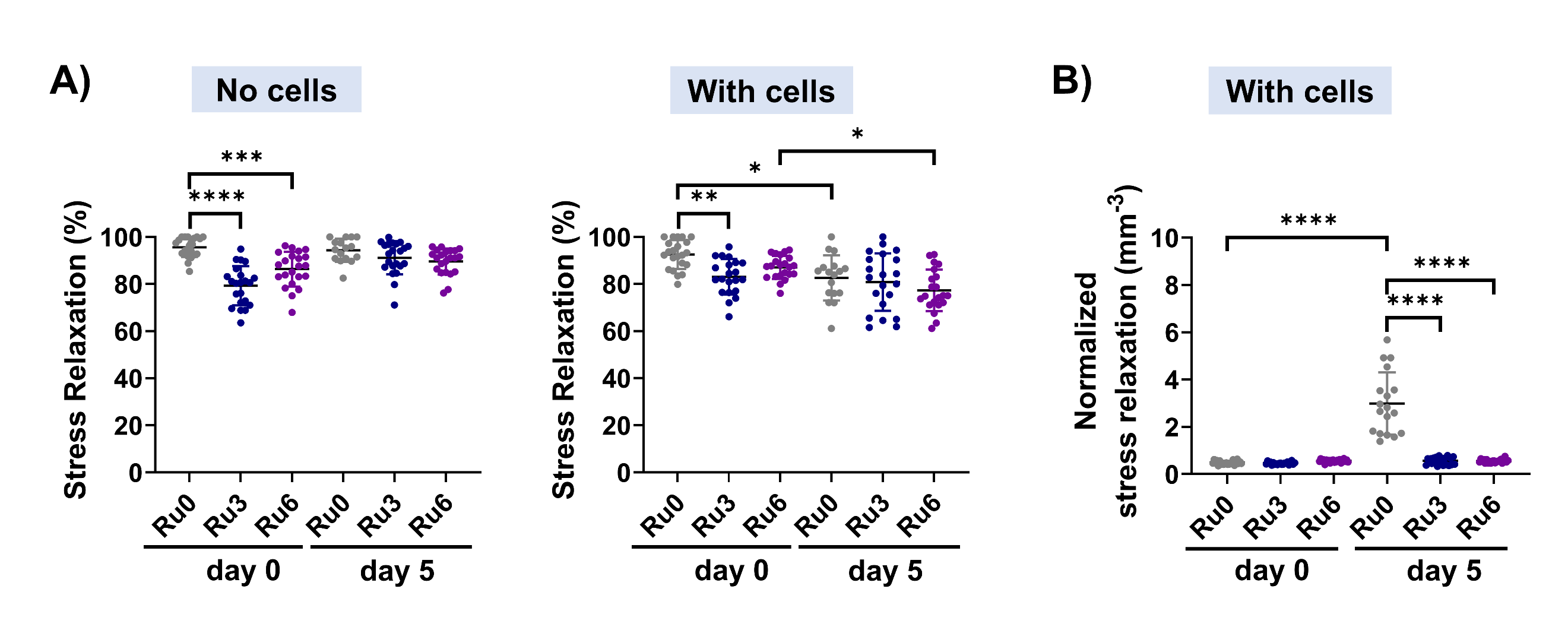


**Fig. S3. The raw and volume-normalized stress relaxation of ECM hydrogels with/without UC-MSCs.** A) Stress relaxation of ECM hydrogels (Ru0, Ru3, and Ru6) at day 0 and day 5 in the absence (left) and presence (right) of UC-MSCs; B) Hydrogel volume-normalized stress relaxation (*SRnorm* = *SRraw*/*V*) of cell-embedded hydrogels at day 0 and day 5, which decouple geometric effects (hydrogel contraction) from intrinsic matrix remodeling. Data are expressed as mean ± standard deviation, with each dot representing technical measurements (two independent tests per hydrogel) from two hydrogel replicates per donor (n = 5). Statistical significance was analyzed by a mixed-effects model using restricted maximum likelihood estimation (REML) (* p < 0.05, ** p<0.01, *** p < 0.001, **** p<0.0001).

**Results: the raw and volume-normalized stress relaxation of ECM hydrogels**

Although the embedded UC-MSCs influenced hydrogel stress relaxation, all three hydrogel groups exhibited stress relaxation values exceeding 77% at both the initial 4-hour time point and after five days of cell culture (Fig. S2). This indicates the rapid stress relaxation and significant viscoelasticity of the ECM hydrogel.

In Ru0 hydrogels, stress relaxation decreased from 92.5 ± 6.1% to 82.6 ± 9.6% (4h vs 5d, P = 0.0056), while Ru6 hydrogels showed a reduction from 87.1 ± 4.9% to 77.3 ± 8.9% (4h vs 5d, P = 0.0033). In contrast, Ru3 hydrogels, which had medium stiffness, exhibited no significant change in stress relaxation (83.1 ± 7.6% vs. 80.8 ± 12.2% for 0d vs 5d, p>0.05). By Day 5, all three hydrogel groups had stress relaxation values within a comparable range (Fig. S2A).

At 0d, Ru0, Ru3, and Ru6 hydrogels displayed similar normalized stress relaxation values (Fig. S2B), consistent with trends observed in the raw stress relaxation data. However, after five days, the decrease in raw stress relaxation observed in Ru6 was no longer evident after normalization, whereas the decrease in Ru0’s raw stress relaxation shifted to an increase in the normalized values. Since stress relaxation is influenced by multiple factors beyond hydrogel contraction-induced changes in matrix density—such as molecular chain mobility, water movement/redistribution, and time-dependent viscoelastic responses—the interpretation of the normalized data remains complex and challenging to fully elucidate.


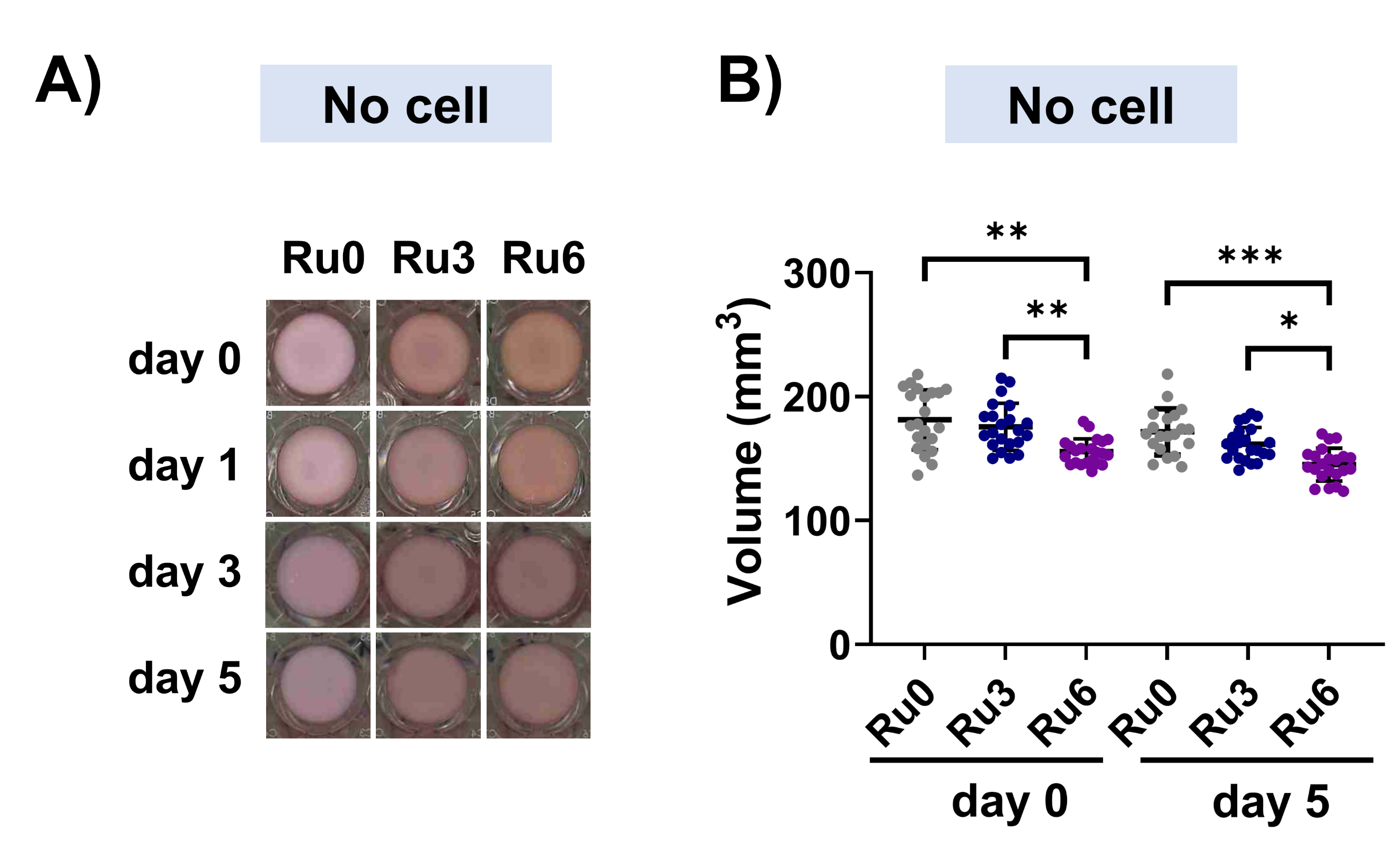


**Fig. S4. Shapes and volumes of cell-free ECM hydrogels.** A) Hydrogel shape (top view): Representative images of the same hydrogels on days 0, 1, 3 and 5; B) Volume (*V*, mm^3^) of cell-free ECM hydrogels on d0 and d5. Data are presented as mean ± standard deviation, with each dot representing technical measurements (2 independent tests per hydrogel) from two hydrogel replicates for each of the UC-MSC donors (n=5). Statistical significance was analyzed by a mixed-effects model using restricted maximum likelihood estimation (REML) (* p < 0.05, ** p<0.01, *** p < 0.001, **** p<0.0001).


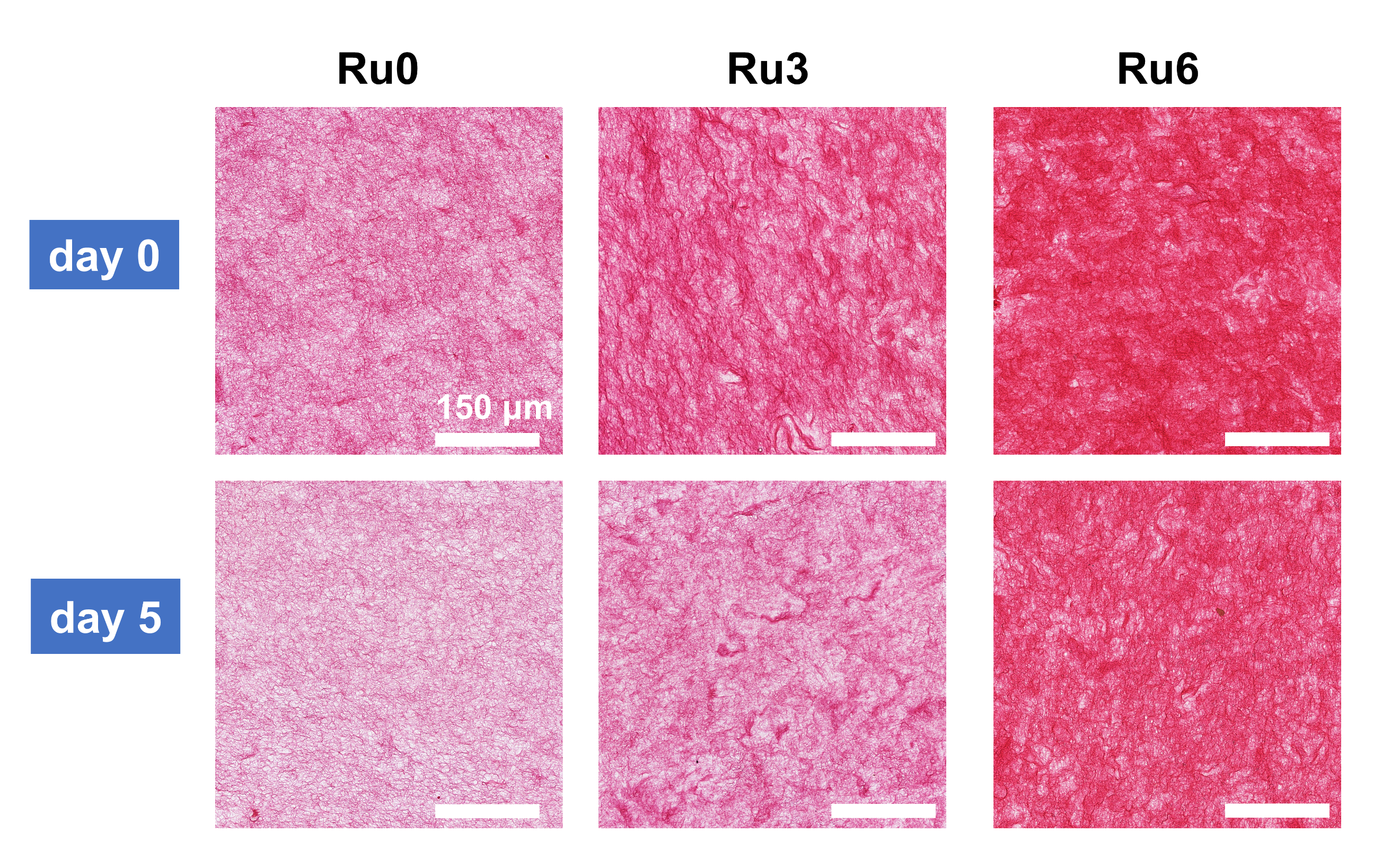


**Fig. S5. Characterization of ECM protein fiber structure in cell-free ECM hydrogels with variable stiffness.** Representative images of PSR staining on 4μm section of non-cell embedded ECM hydrogels (Ru0, Ru3, Ru6) on d0 and d5 (after 5 days of soaking in cell culture medium) (collagen fiber-red). Scale bars:150 μm.


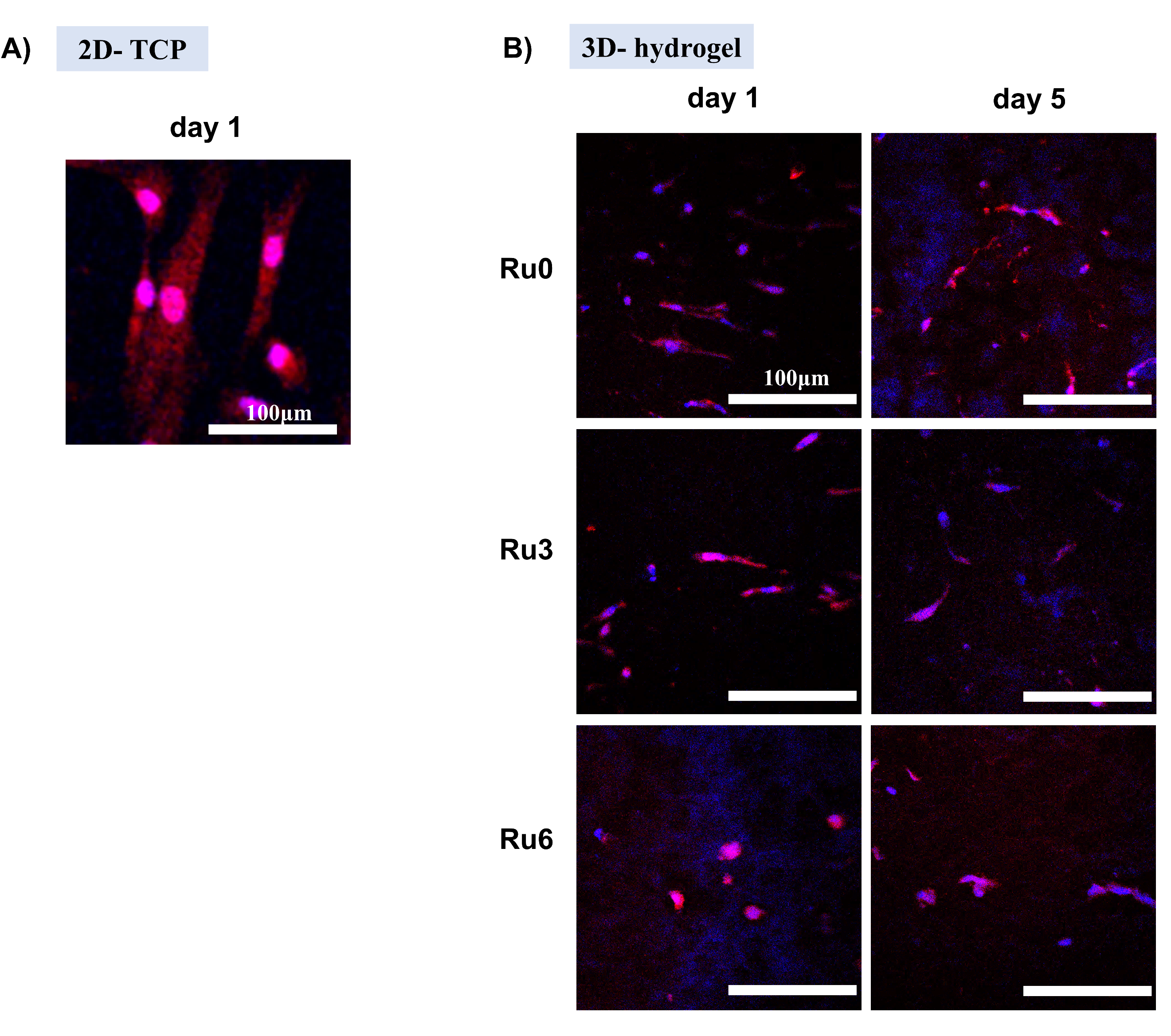


**Fig. S6. YAP expression in 2D and 3D cultured UC-MSCs.** A) Representative immunofluorescence images showing YAP expression (red) in UC-MSCs cultured on TCP plastic (ultra-stiff substrate) and B) in ECM hydrogels (Ru0, Ru3, and Ru6) at day 1 and day 5. Cell nuclei are stained with DAPI (blue).


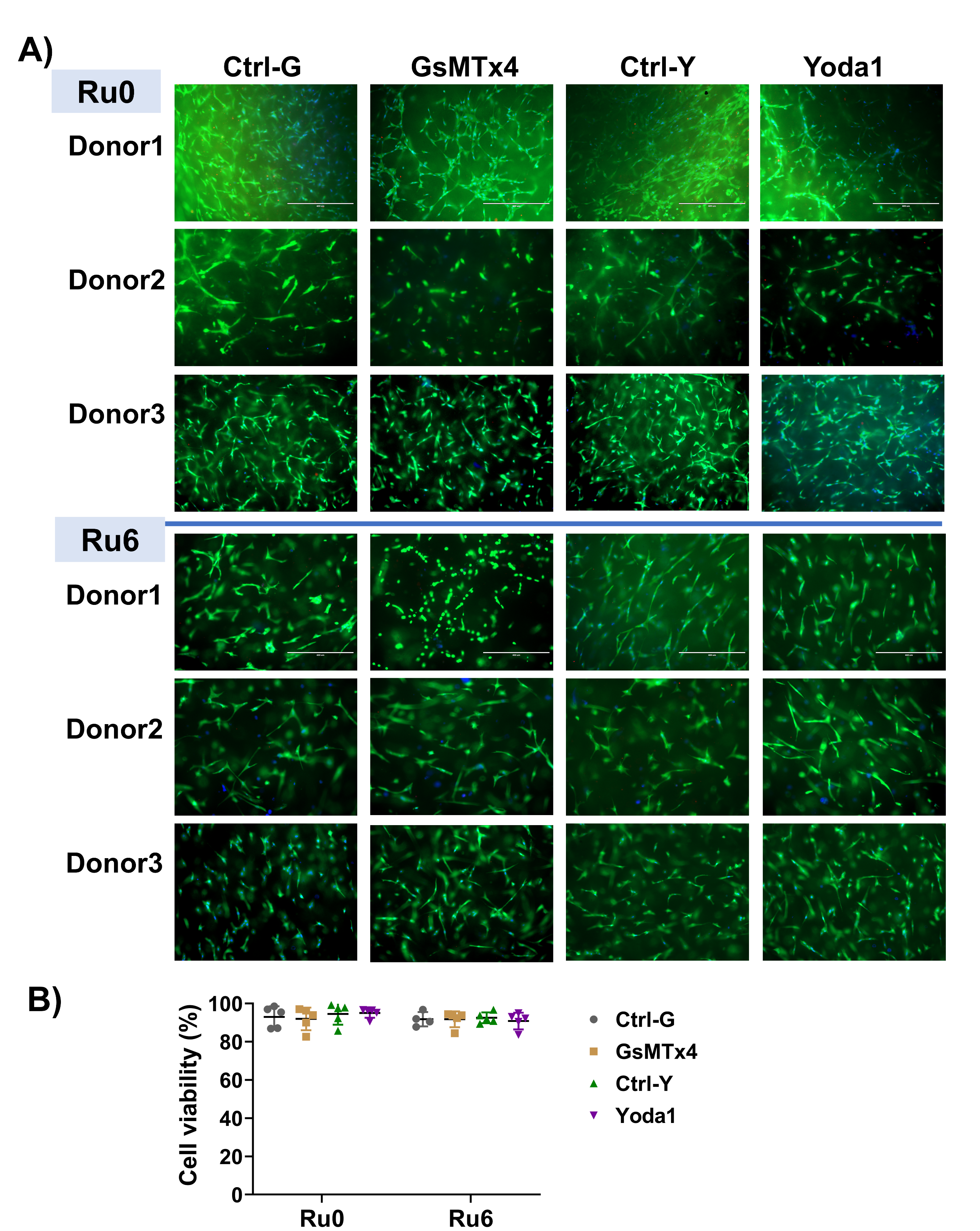


**Fig. S7. The morphology and viability of embedded UC-MSCs in Ru0 and Ru6 ECM hydrogels over 5 days Piezo1 modulation**. A) Representative images showing live-dead and nuclei staining of UC-MSCs in hydrogels. Live cells were stained with Calcein AM (green), nuclei with Hoechst (blue), and dead cells with PI (red). B) Quantification of cell viability, expressed as the percentage (%) of dead cells (PI-positive/DAPI-positive). Data were obtained from two replicate hydrogel samples per donor (n = 3 donors). One image per hydrogel was taken at 10x magnification. Scale bars: 400 μm.


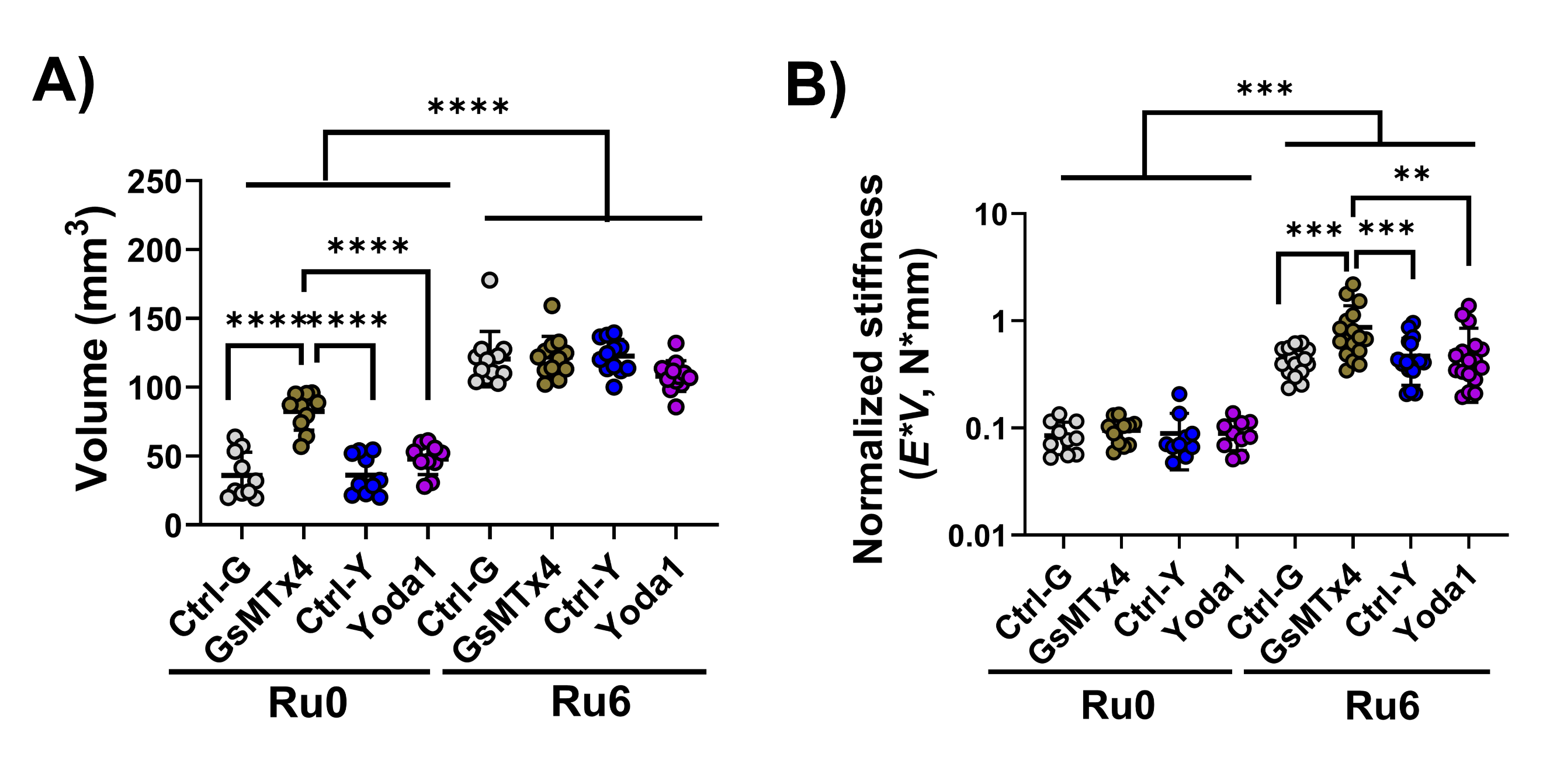


**Fig. S8. Effects of Piezo1 modulation on the volume and volume-normalized stiffness of hydrogels.** A) Hydrogel volume (mm³) of hydrogels at d5 after treatment. B) Volume-normalized stiffness (*E_(norm)_*) at d5, accounting for hydrogel contraction. Data are presented as mean ± standard deviation, with each dot representing technical measurements (2 independent tests per hydrogel) from two hydrogel replicates per cell donor (n=5). Statistical significance was analyzed by a mixed-effects model using restricted maximum likelihood estimation (REML) (* p < 0.05, ** p < 0.01, *** p < 0.001, **** p < 0.0001).


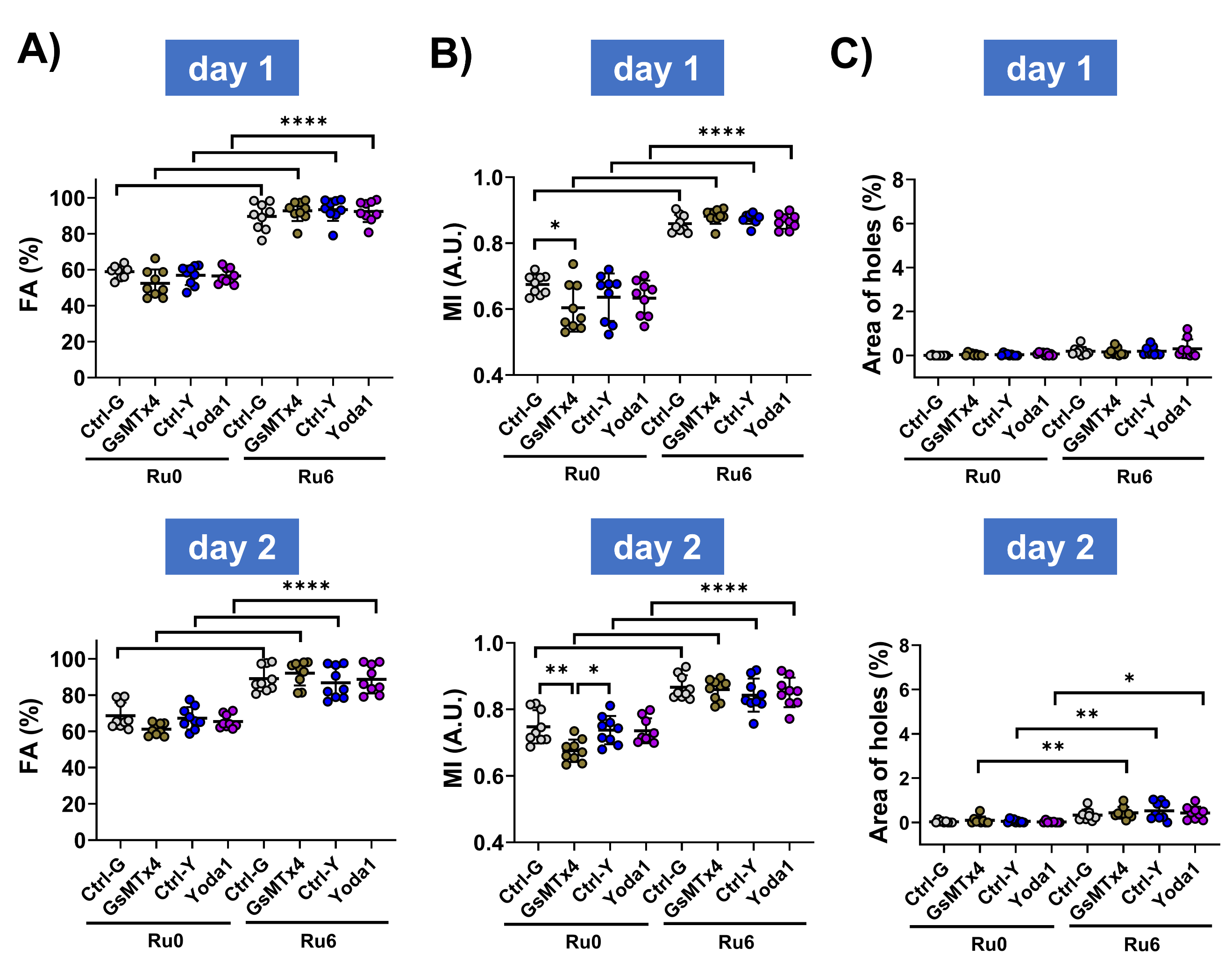


**Fig. S9. Quantitative analysis of collagen matrix remodeling in UC-MSCs-embedded ECM hydrogels after 1d and 2d of Piezo1 modulation.** Fractional area (FA), Mean intensity (MI) and Area percentage of collagen holes surrounding cells in Ru0 and Ru6 group hydrogels across different Piezo1 modulation treatments at day 1 and day 2. FA was calculated by dividing the stained fiber area by the total image area. MI was determined by dividing the total color intensity by the total positively stained fiber area, representing the average PSR staining intensity of collagen fibers. Each dot represents data from three independent measurements of UC-MSC-embedded hydrogels for each cell donor (n = 3). Statistical significance was analyzed by one-way ANOVA (* p < 0.05, ** p<0.01, *** p < 0.001, **** p<0.0001).

**Discussion:**

**Volume normalization to compensate for hydrogel contraction (Continued.)**

Despite the advantages of using linear volume normalization, it may have limitations. For example, Contracted hydrogels (e.g., Ru0, 48% volume loss) exhibit strain-hardening from pore collapse, violating E∝1/V. And, stress relaxation would also be influenced by molecular chain mobility and water redistribution introduce time-dependent effects beyond density. Whatsmore, for collagen fiber staining on 2D section, tight fiber packing in contracted matrices (e.g., Ru0 *FA_raw_* 84.6%) saturates pixel detection, masking true 3D remodeling. Therefore, while linear volume normalization provides useful insights, it should be regarded as a reference rather than definitive evidence of intrinsic ECM remodeling.
